# Elucidating the Respective Roles of Photochemical and Photothermal Effects in Photocatalytic Methanol Decomposition

**DOI:** 10.1007/s40820-026-02305-6

**Published:** 2026-07-27

**Authors:** Qichen Liu, Yida Zhang, Limin Liu, Zixiang Huang, Jiawei Zheng, Shiqin Jian, Haibin Pan, Chi Cao, Hongliang Li, Qing Yang, Yu Bai, Xusheng Zheng

**Affiliations:** 1 National Synchrotron Radiation Laboratory (NSRL), University of Science and Technology of China, Hefei, 230029 Anhui People’s Republic of China; 2 College of Chemistry and Materials Science, University of Science and Technology of China, Hefei, 230026 Anhui People’s Republic of China; 3 College of Chemical Engineering, Inner Mongolia University of Technology, Hohhot, 010051 Inner Mongolia People’s Republic of China; 4 Hefei National Research Center for Physical Sciences at the Microscale, University of Science and Technology of China, Hefei, 230026 Anhui People’s Republic of China; 5 School of Nuclear Science and Technology, Southwest University of Science and Technology, Mianyang, 621010 Sichuan People’s Republic of China; 6 School of Physics and Mechanics, Wuhan University of Technology, Wuhan, 430070 Hubei People’s Republic of China; 7 Experimental Center for Engineering and Materials Science, University of Science and Technology of China, Hefei, 230027 Anhui People’s Republic of China

**Keywords:** Photochemical effect, Photothermal effect, Electron transfer, Synchrotron radiation, H_2_ production

## Abstract

Photocatalytic reactions can be driven by both photochemical and photothermal processes. However, their individual contributions and synergistic effect remain to be clarified. Herein, we investigated the respective roles of these effects using a Cu–TiO_2_/Ti model catalyst that integrated photochemical and photothermal functionalities within a single photocatalytic system. Employing photocatalytic methanol decomposition as a model reaction, the Cu–TiO_2_/Ti catalyst achieved a high H_2_ yield rate of 0.511 mmol h^−1^ with selectivity of 96.6% toward HCHO among oxidation products, attributed to the cooperative action of photochemical and photothermal processes. In situ resonant Auger electron spectroscopy (RAS), together with in situ Cu K-edge X-ray absorption fine structure and excited-state density functional theory calculations, revealed the accumulation of photogenerated electrons at Cu sites from TiO_2_, suggests the possible formation of an electron transfer pathway from TiO_2_ to Cu, which promotes stepwise methanol dehydrogenation to HCHO and H_2_. Simultaneously, the photothermal contribution from Ti enhanced catalytic activity by accelerating reaction kinetics and promoting the desorption of HCHO, thereby suppressing its overoxidation to CO.

**Supplementary Information:**

The online version contains supplementary material available at 10.1007/s40820-026-02305-6.

## Highlights

- A rationally engineered Cu-TiO_2_/Ti model catalyst enables the distinction of photochemical and photothermal effects, revealing their distinct roles in enhancing CH_3_OH dehydrogenation and suppressing HCHO overoxidation, thus achieving high H_2_ production, excellent HCHO selectivity, and long-term stability in a non-noble metal system.
- The integration of in situ resonant Auger electron spectroscopy with in situ diffuse reflectance infrared Fourier transform spectroscopy, in situ Synchrotron radiation photoelectron spectroscopy, and molecular dynamics simulations enable direct visualization of charge transfer pathways and elucidates how the photothermal effect promotes HCHO desorption while preventing overoxidation.

**Supplementary Information:**

The online version contains supplementary material available at 10.1007/s40820-026-02305-6.

## Graphical abstract

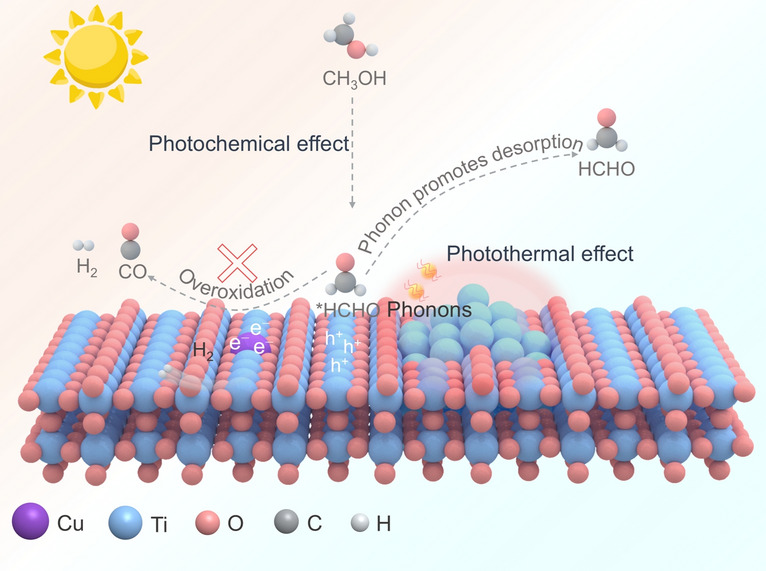

## Introduction

Harnessing sunlight as the sole energy input, photocatalysis holds great promise for energy conversion owing to its sustainability and environmental compatibility. Generally, photocatalytic processes are driven by two primary mechanisms: photochemical and photothermal effects [1, 2]. The photochemical effect involves the generation of hot charge carriers excited by ultraviolet or short-wavelength visible light, which subsequently inject into the antibonding orbitals of adsorbed species [3, 4]. This carrier injection lowers the activation energy barrier, enabling non-thermal reaction pathways [5]. The photothermal effect arises from the absorption of visible and infrared light, where excited electrons undergo non-radiative relaxation back to the ground state [6]. This process releases thermal energy, inducing a localized temperature increase of the catalyst surface that helps overcome reaction energy barriers and drives catalytic conversions [7, 8]. How to synergize carrier injection from the photochemical effect with thermal energy from the photothermal process to overcome the limitations of single-mode mechanisms is of great significance for substantially enhancing photocatalytic performance.

Although the photochemical effect can directly activate substrates, the random recombination of photogenerated charge carriers significantly limits photocatalytic efficiency. Constructing directional electron transfer channels to guide carriers toward active sites is an effective strategy to achieve efficient charge separation and migration [9, 10]. To date, a range of advanced spectroscopic techniques, such as transient absorption spectroscopy (TAS) and time-resolved photoluminescence (TR-PL), have been developed to probe carrier dynamics [11–13]. For instance, Choi et al. employed TAS to characterize the highly efficient interfacial electron transfer in the CdS/CNT photocatalyst, revealing a transfer rate 37-fold faster than that of electron–hole recombination [14]. Zhang et al. demonstrated that introducing Ni_3_S_4_ into ZnCdS significantly accelerated electron transfer using TR-PL, with the formation of a ZnCdS-to-Ni_3_S_4_ electron transport channel facilitating spatial separation of photogenerated carriers [15]. However, these techniques lack elemental and orbital specificity, which can only infer the direction of electron transfer indirectly through accelerated carrier dynamics [16]. Therefore, developing new characterization methods capable of directly observing electron transfer processes in photocatalysis is of critical importance. Herein, we introduce a synchrotron-based resonant Auger electron spectroscopy (RAS) technique. RAS involves electronic transitions to unoccupied states, followed by Auger electron emission from occupied orbitals during the relaxation process [17, 18]. This approach involves the highest occupied molecular orbital (HOMO) and the lowest unoccupied molecular orbital (LUMO) during photocatalytic excitation, providing substantial potential for the precise observation of electron transfer channels.

Current research on the photothermal effect mainly focuses on broadening the solar spectral absorption to increase irradiation-induced temperatures, thereby accelerating photocatalytic reaction rates [19, 20]. For example, Li et al. designed a Bi_2_Te_3_/Cu heterostructure that achieves 89% solar spectrum absorption and successfully heats a CuZnAl catalyst to 305 °C, enabling highly efficient methanol steam reforming for hydrogen production [21]. Liu et al. leveraged the selective absorption properties of a PI/Al/SiO_2_/SiO_2_–Ni metamaterial to effectively reduce thermal radiation losses, significantly enhancing the localized photothermal temperature and thus achieving superior CO_2_ hydrogenation performance [22]. In fact, except for supplying thermal energy to overcome reaction energy barriers, the photothermal effect can also modulate reaction pathways by altering the kinetics of competing reactions and tuning surface species adsorption. Therefore, elucidating the influence of the photothermal effect on product selectivity in photocatalytic systems is also of critical importance.

Here, we constructed a model photothermal catalyst comprising TiO_2_ loaded with Cu single atoms and titanium nanoparticles (Cu–TiO_2_/Ti), which demonstrated the synergistic effect between Cu–TiO_2_ (photochemical unit) and Ti nanoparticles (photothermal unit) during light-driven methanol (CH_3_OH) decomposition for hydrogen (H_2_) production. Specifically, using the developed in situ resonant Auger electron spectroscopy (RAS) combined with in situ Cu K-edge X-ray absorption fine structure (XAFS) and density functional theory (DFT) calculations, we infer a possible electron transfer from TiO_2_ to Cu that promotes stepwise methanol dehydrogenation to HCHO and H_2_, thereby accelerating the stepwise dehydrogenation from CH_3_OH to formaldehyde (HCHO) via hole activation, and promoting H_2_ evolution at Cu^*δ*+^ sites (1 < *δ* < 2) by photogenerated electrons. Mechanistic studies revealed that Ti nanoparticles, serving as the photothermal unit, not only enhanced catalytic activity but also generated phonons to lower the desorption barrier of HCHO, thereby suppressing its overoxidation to carbon monoxide (CO). The synergy of photochemical-photothermal endows the Cu–TiO_2_/Ti catalyst with an impressive H_2_ production rate of 0.511 mmol h^−1^, a high HCHO selectivity of 96.6%, and an outstanding stability exceeding 100 h.

## Experimental Section

### Materials

Ti nanoparticles (Ti NPs), copper (II) chloride dihydrate (CuCl_2_·2H_2_O), sodium sulfate (Na_2_SO_4_), methanol (CH_3_OH), ethanol (C_2_H_5_OH), ethylene glycol (CH_2_OH)_2_, isopropanol (C_3_H_8_O), n-Butanol (C_4_H_10_O), and terpineol (C_10_H_18_O) were purchased from Sinopharm Chemical Reagent Co., Ltd., China. All commercial reagents were used without further purification, and all aqueous solutions were prepared using deionized water with a resistivity of 18.2 MΩ cm^−1^.

### Materials Preparation

#### TiO_2_/Ti

The Ti NPs were heated to 400 °C for 1 h under the atmosphere of air. The obtained powder was ground to obtain the TiO_2_/Ti.

#### TiO_2_

The Ti NPs were heated to 800 °C for 1 h under the atmosphere of air. The obtained powder was ground to obtain the TiO_2_ material.

#### Cu–TiO_2_/Ti

For the synthesis of Cu–TiO_2_/Ti, TiO_2_/Ti (200 mg) was dispersed in a mixed solution of CuCl_2_ water solution (20 mL, 5.3 mM) and C_2_H_5_OH (40 mL). Then, the obtained suspension was stirred for 4 h, centrifuged, and washed with water to obtain Cu–TiO_2_/Ti.

### Structural Characterizations and First‑Principles Calculations

#### Photocatalytic Test

The photocatalytic reactions were conducted in a batch quartz reactor. Each reaction was performed in 20 mL of CH_3_OH by using 200 mg of catalysts under the illumination of a 300 W Xenon lamp (PLS-SXE300E, Beijing Perfectlight Technology Co., Ltd.) at room temperature. During the photocatalytic experiments, the light intensity at the reaction system was maintained at 100–500 mW cm^−2^ by adjusting the lamp current, as measured with an optical power meter (MC-PM100B, Beijing Merry Change Technology Co., Ltd.). The generated H_2_ and CO were detected by gas chromatography (East & West Analytical Instruments, Inc., GC-4400). HCHO product was detected by the acetylacetone colorimetric method using a UV–vis spectrophotometer (Cary 60 UV–Vis) [23]. Proton nuclear magnetic resonance (1H NMR) spectra were obtained to qualitatively determine the products on a Bruker- AVANCE III 400 MHz instrument.

#### RAS Measurement

The RAS tests were conducted at BL10B (31,131.02.HLS.PES) of NSRL, using the EW4000 electron energy analyzer to collect and analyze electrons. The beamline monochromator was connected to SES software (Scienta Omicron, version 1.8.0) for synchronous control of incident photon energy during electron detection. As for Ti RAS measurement, the incident photon energy was 452–470 eV, and the kinetic energy scanning range was 411–427 eV. In a typical Cu RAS measurement, the incident photon energy was 932–940 eV, and the kinetic energy scanning range was 913–925 eV. The spectra were collected under darkness or light irradiation. The frames of the charge-coupled device were set as 160 [24].

#### X-Ray Absorption Fine Structure (XAFS) Measurement

The XAFS spectra of Ti K-edge (*E*_0_ = 4966 eV) and Cu K-edge (*E*_0_ = 8979 eV) were measured at beamline BL14W1 (31,124.02. SSRF.BL14W1) and BL11B (31,124.02. SSRF.BL11B) of Shanghai Synchrotron Radiation Facility (SSRF). The Ti K-edge spectra were recorded under transmission mode with two ion chambers. The Cu K-edge spectrum of Cu–TiO_2_/Ti was recorded under fluorescence mode with a Lytle chamber. Athena and Artemis codes were used to analyze the data. The parameters described the local structure environment including coordination numbers (*CN*), bond distance (*R*), and Debye–Waller (*DW*) factor around the absorbed atoms were allowed to vary during the fit process [25].

#### Synchrotron Radiation Photoelectron Spectroscopy (SRPES) Measurements

The SRPES measurements were performed at the photoemission end-station at beamline BL10B (31,131.02.HLS.PES) in the National Synchrotron Radiation Laboratory (NSRL) in Hefei, China. The beamline is connected to a bending magnet and covers photon energies from 100 to 1000 eV with a resolving power (E/*Δ*E) better than 1000. The end-station is composed of four chambers—an analysis chamber, a preparation chamber, a load-lock chamber, and a high-pressure reactor. Copper tubing at the base of the sample holder achieves low temperatures via a condenser to suppress the photothermal effect, while a thermocouple mounted on the surface enables real-time monitoring of the holder’s temperature. The analysis chamber, with a base pressure of < 2 × 10^−10^ torr, is connected to the beamline with an EW4000 electron energy analyzer (Scienta Omicron) and a twin anode X-ray source. The high-pressure reactor contains a reaction cell where the samples can be treated with different gases up to 20 bar and simultaneously with light irradiation. After the sample treatment, the reactor can be pumped with pressure down to < 10^−8^ torr for sample transfer [26].

In this work, the sample was treated with 1 bar of argon, which bubbled in CH_3_OH and then transferred to the analysis chamber for XPS measurement without exposure to the atmosphere.

#### Photoelectrochemical Measurement

Electrochemical impedance spectroscopy (EIS) and photocurrent response measurements were performed using a CHI 660E electrochemical workstation (Chenhua Instruments, Shanghai, China). The FTO glasses were first cleaned by ultrasonic with acetone, ethanol, and H_2_O for 30 min, respectively. Then, 5.0 mg of the samples was dispersed into 0.5 mL of terpineol by ultrasonic. The as-obtained suspension was dropped onto a 1 cm × 1 cm FTO glass, and then the FTO glass was dried out overnight in an oven at 80 °C. Pt foil and Ag/AgCl electrodes were used as the counter and reference electrodes, respectively. 0.5 M Na_2_SO_4_ aqueous solution was used as the electrolyte.

#### In Situ Diffuse Reflectance Infrared Fourier Transform Spectroscopy (DRIFTS) Measurement

In situ DRIFTS tests were performed at the beamline BL01B (31,131.02.HLS.IRSM) of NSRL. The Bruker IFS 66v Fourier-transform spectrometer equipped with a Harrick diffuse reflectance accessory. The in situ infrared device enables introducing light irradiation in a sealed CH₃OH atmosphere, while controlling the system temperature through a condenser and a thermocouple. Each spectrum was recorded by averaging 128 scans at a 6 cm^−1^ spectral resolution [27].

#### Instrumentations

SEM images were collected on an FEI XL-30 ESEM scanning electron microscope. HRTEM images were recorded using a Talos F200X high-resolution transmission electron microscope. XRD patterns were collected by TTR Ⅲ theta-theta rotating anode X-ray diffractometer using Cu-Kα radiation (*λ* = 1.54178 Å). The photoluminescence (PL) spectra were measured using a steady-state/lifetime spectrofluorometer (Fluorolog-3-Tau and DeltaFlex). The measurements were carried out at room temperature using a 325 nm excitation wavelength, and the emission spectra were collected in the range of 370–520 nm. The UV–vis diffuse reflectance spectra were recorded on a UV–visible-near infrared Spectrophotometer (Solid 3700 DUV). The loading of Cu was determined by inductively coupled plasma-atomic emission spectrometry (ICP-AES, iCAP 7400).

#### Calculation Details

All calculations were performed with the density functional theory (DFT) implemented in the Vienna Ab initio Simulation Package [28]. The ion–electron interaction is described by the projector augmented wave potentials. Spin-polarized Perdew–Burke–Ernzerhof functional of the generalized gradient approximation was employed to treat the exchange correlation between electrons [29]. The Cu–TiO_2_(110) model was constructed by the Cu single atom loaded TiO_2_ (110) facet. A vacuum slab of 15 Å thickness along the c direction was applied to separate the interactions between neighboring images [30]. During the calculations, the cutoff energy was set as 500 eV, and the total energy convergence was set to be lower than 1 × 10^−5^ eV with the force convergence set at 0.02 eV Å^−1^. Brillouin zone is sampled with a k-point mesh of 3 × 2 × 1. The D3 method with Becke-Johnson damping function was utilized to improve the description of van der Waals interactions. Considering the electron correlations in the TiO_2_ crystal, GGA+U method was explicitly taken into account, and Hubbard on-site Coulomb parameter Ueff was 4.2 eV for Ti atoms [31]. To simulate electronic excitation from the ground state to the excite state, the occupation constrained DFT calculations were carried out, in which one electron was placed from the highest occupied band to the lowest unoccupied band. For the free energy variations calculation, we conducted a computational hydrogen electrode model according to the expression by VASPKIT with the consideration of spin polarization: *ΔG* = *ΔE* + *Δ*ZPE–T*Δ*S + *ΔU*(0 → T), where *Δ*E denotes adsorption energy, *Δ*ZPE represents zero-point energy, *Δ*S corresponds to entropy change at a temperature of 298 K, and *ΔU*(0 → *T*) means the thermal correction to the internal energy. Zero-point energy and entropy for adsorbents were determined by vibrational frequency calculations using density functional perturbation theory, while entropy values for gaseous species were sourced from the Computational Chemistry Comparison and Benchmark Database.

The constrained molecular dynamics (MD) simulations were performed by employing the VASP package. The MD simulations were sampled by the canonical (NVT) ensemble employing Nose–Hoover thermostats with a time step of 1.0 fs at the target temperature of 293 K and 333 K. In the thermodynamic integration (TI) method, the reaction free energy and kinetic barrier were obtained by applying a holonomic constraint on the reaction coordinate (ζ) during MD simulations and integrating over the average unbiased force associated with the reaction coordinate, as shown in Eq. 1:

1 $$ \Delta E\left( {\zeta_{a} ,\zeta_{b} } \right) = - \int_{{\zeta_{a} }}^{{\zeta_{b} }} {F\left( \zeta \right)d\zeta } $$

where $\Delta E\left( {\zeta_{a} ,\zeta_{b} } \right)$ is the free energy difference between two reaction coordinates $\left( {\zeta_{a} \, and \, \zeta_{b} } \right)$ and $F\left( \zeta \right)$ is the averaged constrained force. For the HCHO desorption process on the Ti site, the sum of Z-axis coordinates over H, C, H, and O atoms in HCHO is chosen as the collective variable (CV), which is defined by Eq. 2:

2 $$ {\text{CV }} = \, Z_{{\mathrm{H}}} + \, Z_{{\mathrm{C}}} + \, Z_{{\mathrm{H}}} + \, Z_{{\mathrm{O}}} $$

where Z_H_, Z_C_, Z_H_, and Z_O_ refer to Z-axis coordinates over H, C, H, and O atoms in HCHO, respectively [32].

## Results and Discussion

**Fig. 1:**
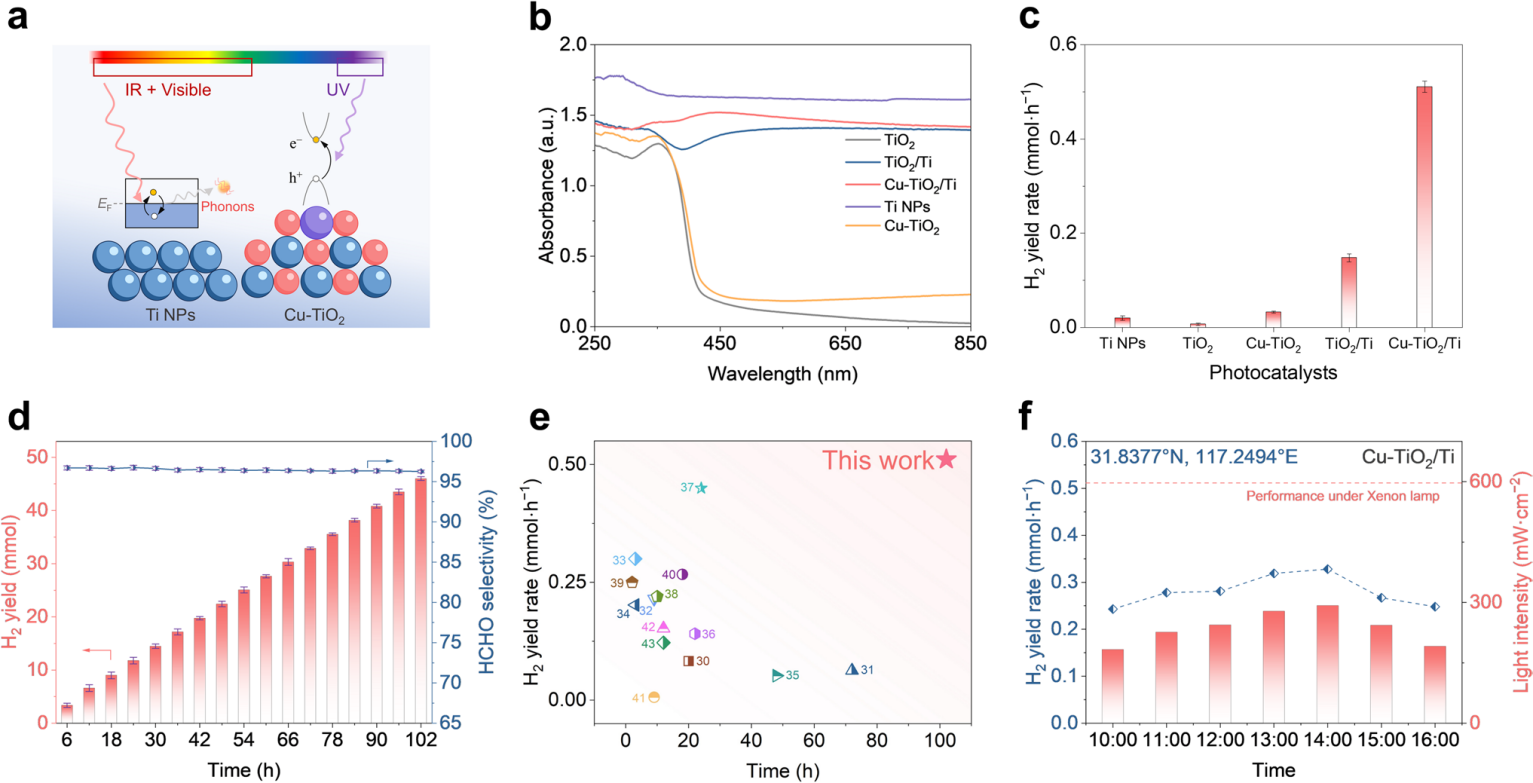
**a** Role and response wavelength of each unit in Cu-TiO_2_/Ti. **b** UV–vis spectra for Ti NPs, TiO_2_, TiO_2_/Ti, Cu-TiO_2_ and Cu-TiO_2_/Ti samples. **c** H_2_ yield rates over Ti NPs, TiO_2_, Cu-TiO_2_, TiO_2_/Ti, and Cu-TiO_2_/Ti samples, respectively (Light intensity: 500 mW cm^−2^). **d** Time-dependent yield of H_2_ and selectivity of HCHO over Cu-TiO_2_/Ti. The error bars denote standard deviations from three independent experiments. (Light intensity: 500 mW cm^−2^) **e** Comparison of the stability and H_2_ yield rate of Cu-TiO_2_/Ti catalyst with those of the reported catalysts. **f** H_2_ yield rates over Cu-TiO_2_/Ti under actual sunlight illumination and real-time solar irradiance

To elucidate the synergistic effects of photochemical and photothermal processes during photocatalytic reaction, we designed a photochemical-photothermal coupling system (Fig. 1a). The photochemical effect is driven by photogenerated charge carriers from TiO_2_ upon ultraviolet excitation, enabling initiation of the reaction via direct activation of substrate molecules. The photothermal effect, on the other hand, is realized through the absorption of visible and infrared light by Ti nanoparticles (NPs), which generates phonons via a non-radiative relaxation process. Besides, the introduction of a cocatalyst is also crucial for achieving efficient photocatalytic processes. Therefore, a model photocatalyst consisting of Cu cocatalyst supported on TiO_2_/Ti was developed (Cu–TiO_2_/Ti).

Cu–TiO_2_/Ti composite was synthesized through a one-step calcination, followed by impregnation to load Cu cocatalyst (see details in Supplementary Information). High-resolution transmission electron microscopy (HRTEM) images displayed two sets of fringes with interplanar spacing of 0.33 and 0.25 nm, corresponding to the (110) plane of rutile TiO_2_ and the (100) plane of metallic Ti, respectively (Fig. S1) [33]. X-ray diffraction (XRD) patterns indicated the presence of TiO_2_ in the rutile phase and Ti in the alpha phase (Figs. S2 and S3). No characteristic peaks of Cu species, such as Cu, Cu_2_O, or CuO, were observed. Energy dispersive spectroscopy (EDS) elemental mapping images showed that Cu species were uniformly distributed over the TiO_2_/Ti support (Fig. S4). The loading amount of Cu was determined as 0.45 wt% by inductively coupled plasma-atomic emission spectrometry (ICP-AES, Table S1). To further investigate the precise structure of the as-obtained sample, X-ray absorption fine structure (XAFS) measurements were conducted. As shown in Fig. S5, the absorption edge of Cu–TiO_2_/Ti was between those of Ti NPs and TiO_2_, indicating that the average oxidation state of Ti species was less than + 4, which was consistent with X-ray photoelectron spectroscopy (XPS) results (Fig. S6) [34, 35]. Linear combination fitting analysis revealed that the proportion of the Ti and TiO_2_ content in the Cu–TiO_2_/Ti sample was approximately 60% and 40%, respectively (Fig. S7) [36]. Fourier-transform extended X-ray absorption fine structure (FT-EXAFS) measurements were performed to determine the local coordination environment of Cu. As depicted in Figures S8 and S9, Cu–TiO_2_/Ti displayed a prominent peak at 1.5 Å, attributed to Cu–O coordination. The absence of Cu–Cu scattering pathway suggested that Cu existed as isolated single atoms on TiO_2_ (Table S2) [37, 38]. Additionally, HAADF-TEM characterization was performed on the Cu–TiO_2_/Ti sample, where isolated bright spots corresponding to individual Cu atoms were observed, further confirming that Cu exists in the form of single atoms (Fig. S10).

To investigate the solar light absorption capabilities of different components in the photocatalyst, the optical properties of each component were analyzed using ultraviolet–visible (UV–vis) spectra (Fig. 1b). The TiO_2_ and Cu–TiO_2_ primarily absorb ultraviolet light and serve as the elementary material for the photochemical effect in photocatalysis. The introduction of Cu into the TiO_2_ substrate results in a redshift of the absorption edge and enhanced light absorption in the 450–850 nm range, suggesting that the incorporation of Cu may introduce impurity energy levels, thereby promoting photon absorption. The Ti nanoparticle achieves full-spectrum absorption, demonstrating an efficient photothermal effect. The TiO_2_/Ti composite combines the capabilities of TiO_2_ and Ti NPs, exhibiting full-spectrum absorption from 250 to 850 nm. This indicates that the prepared photothermal catalyst can absorb the entire solar spectrum. Further introducing Cu into the TiO_2_/Ti substrate enhances photon absorption around 450 nm. This improvement is attributed to Cu doping, which introduces impurity energy levels that facilitate more efficient photon absorption [39].

The photocatalytic hydrogen production from methanol was employed as a model reaction to evaluate the synergy between the photochemical and photothermal contributions of the Cu–TiO_2_/Ti photocatalyst. Specifically, the photocatalytic H_2_ production rates of the obtained samples were evaluated. Each reaction was performed in 20 mL of CH_3_OH under the irradiation of a 300 W Xenon lamp (Fig. S11). As displayed in Fig. 1c, the utilization of Ti NPs or TiO_2_ samples as catalysts resulted in negligible H_2_ production. Upon coupling Ti and TiO_2_, the yield rate of H_2_ significantly increased to 0.14 mmol h^−1^, which could be attributed to the synergy of photochemical effect and photothermal effect (Fig. S12). The further incorporation of Cu single atoms efficiently promoted the photochemical process, which achieved a superior H_2_ yield rate of 0.511 mmol h^−1^ and a selectivity of 96.6% for formaldehyde (HCHO) among oxidation products over Cu–TiO_2_/Ti (Fig. S13, Table S3). Meanwhile, the solar-to-chemical conversion efficiency (SCC) of the Cu–TiO_2_/Ti catalyst under 1 sun AM 1.5G illumination for photocatalytic hydrogen production from methanol is 0.21% (Table S4), and the apparent quantum yield (AQY) under 365 nm LED light is 15.9%, which is 3.7 times that of TiO_2_/Ti (4.3%) (Table S5).

Besides, a remarkable catalytic stability of 102 h was also obtained over Cu–TiO_2_/Ti (Fig. 1d), which ranked among the best non-noble metal-based catalysts for H_2_ production performance from CH_3_OH, even comparable to the reported Pt-based noble metal catalysts (Fig. 1e, Table S6) [40–53]. To check the stability of the catalyst, we collected the XRD pattern, SEM image, and Cu K-edge XAFS data for Cu–TiO_2_/Ti after reactions. As displayed in XRD spectra, the crystal structure of Cu–TiO_2_/Ti was maintained after reactions (Fig. S14a). Meanwhile, no typical Cu nanoparticles or clusters were observed. This point was further confirmed by the SEM image of Cu–TiO_2_/Ti after reactions (Fig. S14b). Furthermore, we collected Cu K-edge XAFS spectra to investigate the chemical states and atomic structure of Cu single atoms. Apparently, the Cu species in Cu–TiO_2_/Ti inherited the initial oxidized state and atomic dispersion after reactions (Figs. S14c, d, Table S2), further indicating the remarkable stability of Cu–TiO_2_/Ti. Moreover, outdoor experiments were conducted to evaluate the photocatalytic hydrogen production potential of this coupling system under practical conditions. As illustrated in Fig. 1f, Cu–TiO_2_/Ti catalyst obtained a hydrogen evolution rate exceeding 0.20 mmol h^−1^ during natural sunlight irradiation from 10:00 to 16:00. When solar irradiance approached its peak, the system achieved an optimal hydrogen production rate of 0.33 mmol h^−1^ at 14:00, which was 64.7% of that using a xenon lamp. Importantly, the photocatalytic performance under real sunlight was further compared with that obtained under xenon lamp irradiation at different controlled light intensities (Figs. S14–S17). A good correlation between the two datasets was observed, indicating that the performance under natural sunlight could be well matched with that under simulated solar irradiation at equivalent light intensities. pansion experiments using different alcoh.

**Fig. 2:**
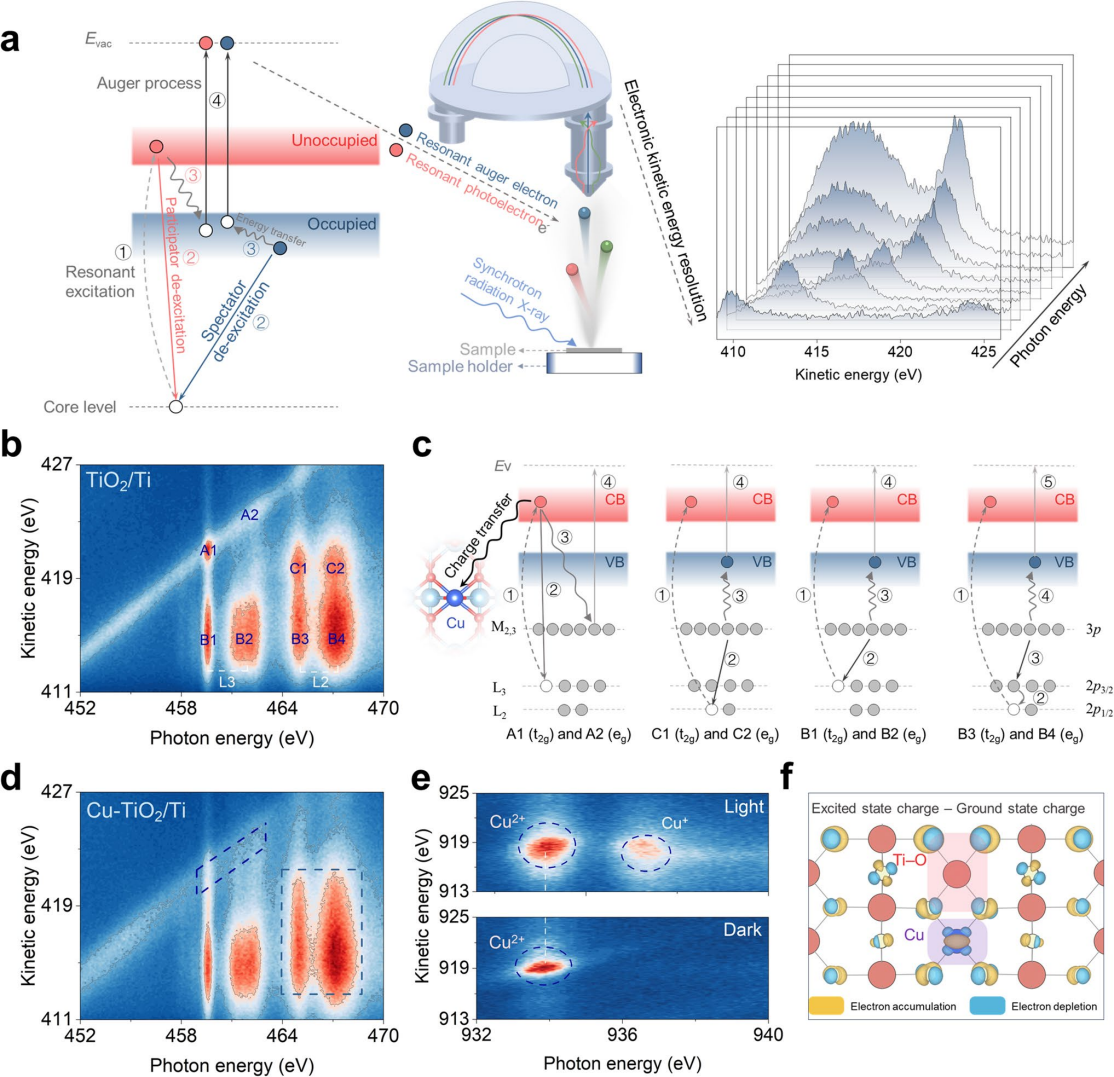
**a** Measurement principle and process of resonant Auger electron spectra. **b** Ti RAS spectrum of TiO_2_/Ti. **c** Schematic diagram of electron transfer process from RAS tests. **d** Ti RAS spectrum of Cu-TiO_2_/Ti. **e** In situ Cu RAS spectra of Cu-TiO_2_/Ti. **f** Charge density difference of Cu-TiO_2_(110) model under the excited state. The balls in light yellow, red, and blue represent O, Ti, and Cu atoms, respectively. The yellow and cyan colors represent electron accumulation and depletion, respectively

To elucidate the mechanistic processes of the photochemical effect in the Cu–TiO_2_/Ti system, we performed in situ resonant Auger electron spectroscopy (RAS) tests. Benefiting from the photon energy tunability of synchrotron radiation facility, RAS measurements employ an energy analyzer to collect Auger electrons with specific kinetic energy at each resonant energy, thus obtaining a two-dimensional resonant Auger electron map (Fig. 2a). In this process, a core-level electron is first excited to unoccupied orbitals. The system then relaxes via two competing channels: participator de-excitation, in which the excited electron fills the core hole, and spectator de-excitation, in which another electron from an occupied orbital fills the core hole while the excited electron remains in the unoccupied state. These de-excitation processes result in the emission of Auger electrons from occupied orbitals [16]. As a result, RAS simultaneously probes both unoccupied and occupied states through the initial core excitation and subsequent Auger emission, which offers an orbital-level and element-specific insight into the electronic structure. This capability makes RAS particularly powerful for investigating photocatalytic electron behaviors involving transitions from HOMO to LUMO.

As illustrated in Fig. 2b, the Ti RAS spectrum of TiO_2_/Ti exhibited several distinct features labeled A, B, and C. Feature A corresponded to participator de-excitation, while features B and C were attributed to spectator de-excitation pathways (Figs. 2c and S18). Compared to TiO_2_/Ti, the RAS features of Cu–TiO_2_/Ti displayed pronounced broadening along both photon energy (horizontal) and kinetic energy (vertical) axes (marked by blue rectangles). This observation reflected the simultaneous broadening of the HOMO and LUMO levels, deriving from the incorporation of Cu single atoms (Fig. 2d). Such band broadening optimized the optical properties of Cu–TiO_2_/Ti that enabling it to absorb photons across a wider energy range, which further proved UV–vis results. Notably, the Cu–TiO_2_/Ti system exhibited a marked decrease in the proportion of participator decay (feature A) relative to TiO_2_/Ti, decreasing to approximately 50% of that of TiO_2_/Ti. Quantitative analysis of the Ti RAS spectra (Table S7) shows that the participator decay (feature A) accounts for 14.4% of the total spectral weight in TiO_2_/Ti, but decreases to only 7.5% in Cu–TiO_2_/Ti. Concurrently, the spectator decay (feature B) increases from 85.6 to 92.5%. This observation suggested that the excited-state electrons promoted to the unoccupied states were less likely to undergo de-excitation and recombine with holes. Instead, these electrons were inferred to be more readily transferred to surrounding active sites, thereby participating in the reaction process [54, 55]. Thus, we propose that the incorporation of Cu modifies the electronic structure, leading to redistribution of photoexcited electrons. This point was further verified by in situ Cu RAS spectra of Cu–TiO_2_/Ti (Fig. 2e and c). Compared with the dark condition, the signal of Cu^+^ species significantly enhanced under light, indicating that photogenerated electrons that originally enriched in the Ti 3*d* states converted to the Cu sites (Fig. S19) [56, 57]. Quantitative fitting of the Cu RAS spectra (Table S8 and Table S9 reveals that under dark conditions, Cu exists almost exclusively as Cu^2+^ (~ 100%). Upon light irradiation, a substantial fraction of Cu^2+^ is reduced to Cu⁺, with Cu⁺ accounting for 41.0% of the total Cu signal. In contrast, under thermal treatment (343 K in darkness), no Cu⁺ is detected, confirming that the reduction is photoinduced rather than thermally driven (Table S9). Additionally, in situ XAFS measurements further support this conclusion. In situ Cu K-edge spectra, a shift of the Cu K-edge absorption edge toward lower energy was observed upon illumination, indicating a decrease in the oxidation state of Cu (Fig. S19a, b). This result further confirms that photogenerated electrons are transferred to and accumulated at the Cu sites. As a result, Cu^*δ*+^ (1 < *δ* < 2) species were obtained upon photon excitation in the Cu–TiO_2_/Ti catalyst. This electron transfer channel facilitates the spatial separation and efficient transport of photogenerated electrons, which was further confirmed by PL, transient photocurrent response, and electrochemical impedance spectra under light (Figs. S20–S23, Table S10) [58–60]. Meanwhile, in situ RAS and XAFS measurements under dark thermal conditions show results highly consistent with the ex situ data, indicating that the photothermal effect does not induce noticeable changes in the electronic structure. These results demonstrate that the Cu active sites do not undergo detectable electronic or atomic structural changes under purely thermal conditions, further supporting that the catalytic activity originates primarily from the photochemical effect (Fig. S19).

To validate this point, differential charge density calculations were further conducted. We established a catalyst model, denoted as Cu–TiO_2_(110), featuring a Cu single atom loaded on the TiO_2_ (110) facet. The DFT calculations reveal that Cu incorporation into TiO_2_(110) introduces a mid-gap state mainly derived from Cu and adjacent O orbitals, which acts as an efficient electron-trapping site. This promotes directional electron transfer from Ti 3*d* states to Cu sites, leading to electron accumulation at Cu active centers (Fig. S24). For simulating photoexcitation, one electron was removed from the HOMO and placed at the LUMO (Fig. S25) [61]. For clarity, the electronic configuration before and after this adjustment was denoted as the ground state and excited state, respectively. As shown in Fig. 2f, the differential charge density revealed electron depletion (holes) localized at Ti sites surrounding the Cu atom (marked by red rectangles), and electron accumulation at the Cu site (marked by purple rectangles). Besides, the H_2_ production pathway was further screened. The Gibbs free energy change for H_2_ generation over the Cu site was determined as 0.76 eV in the ground state. When it comes to the excited state, this Gibbs free energy change decreased to 0.38 eV. Therefore, the excellent photochemical effect of Cu–TiO_2_/Ti was attributed to the efficient separation of photogenerated carriers under photoexcitation, with holes localized at Ti sites and electrons accumulated at Cu sites. Also, the in situ generated Cu^*δ*+^ (1 < *δ* < 2) species promoted the H_2_ production via lowering the reaction energy barrier (Fig. S25).

**Fig. 3:**
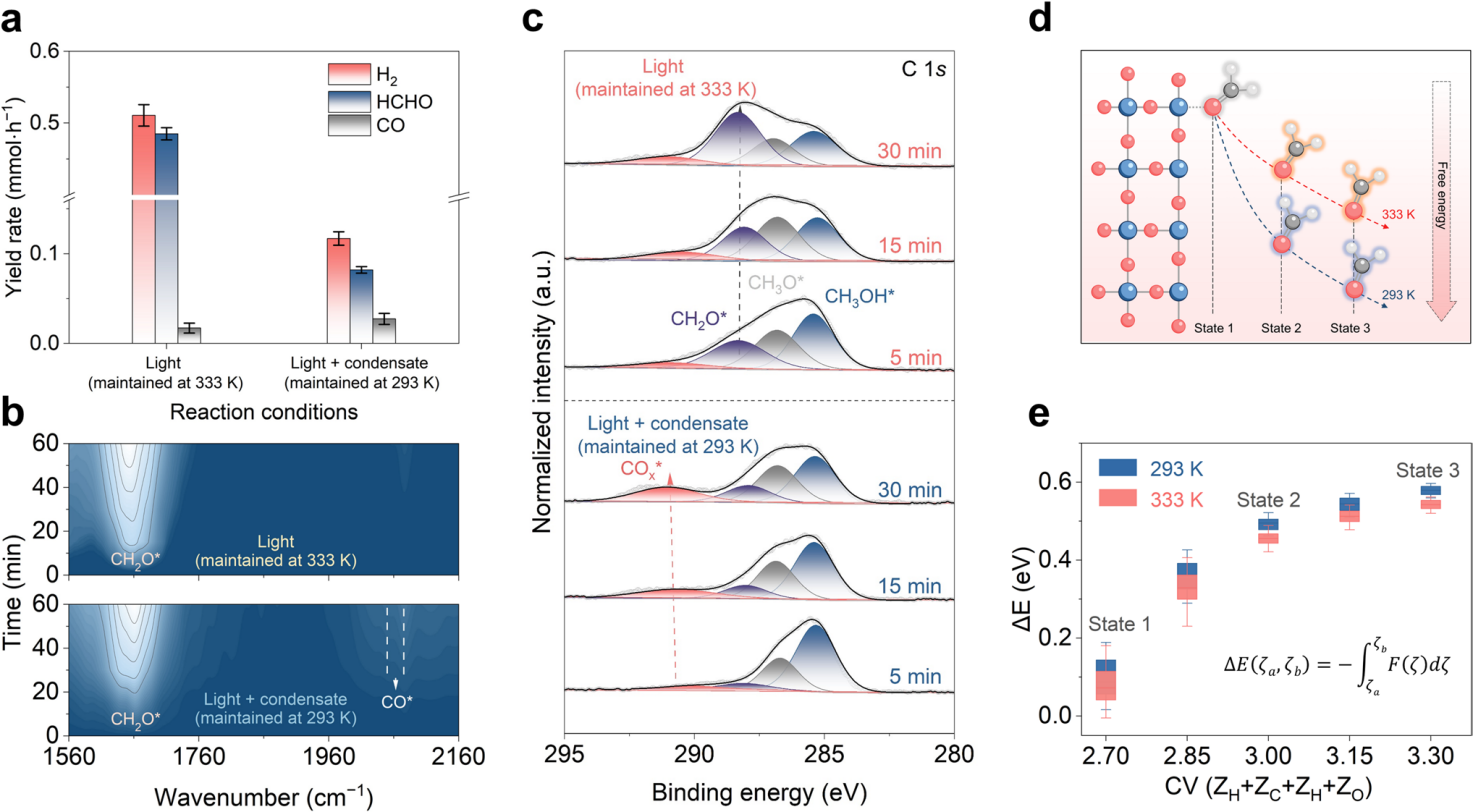
**a** The yield rates of H_2_, HCHO, and CO over Cu-TiO_2_/Ti under different reaction conditions (Light intensity: 500mW cm^−2^). **b** In situ DRIFTS spectra over Cu-TiO_2_/Ti under light (above) and (below) light with condensate. **c** In situ C 1*s* SRPES spectra under light (above) and light with condensate (below) over Cu-TiO_2_/Ti, respectively. **d** Theoretical models of HCHO desorption over Cu-TiO_2_(110) model under different collective variable conditions. **e** Potential-dependent free energies of the HCHO desorption process over Cu-TiO_2_(110) model surface at 333 K and 293 K, respectively. *ΔE*(ζ_a_, ζ_b_) is the free energy difference between two reaction coordinates. (ζ_a_ and ζ_b_) and F(ζ) is the averaged constrained force

After clarifying the photochemical effect in the Cu–TiO_2_/Ti photocatalytic system, we turned our attention to decoupling the contribution of the photothermal effect. We first monitored the real-time temperature of CH_3_OH liquid over different samples under illumination. As displayed in Fig. S26, the temperature of the Cu/TiO_2_ system increased slightly from 293 to 304 K. When it comes to Cu–TiO_2_/Ti and TiO_2_/Ti, the temperature rose rapidly to 333 K within 180 s, attributable to the photothermal conversion capability of metallic Ti. This conclusion was further corroborated in the photocurrent response measurements, a pronounced increasing trend in the slow response current (SRC) component of the photocurrent was observed for the Cu–TiO_2_/Ti, indicating a significant photothermal effect and a corresponding temperature rise in the testing system. In contrast, no obvious photocurrent increasing trend was detected in the SRC component of the Cu–TiO_2_, suggesting that the photothermal effect was negligible (Fig. S27). These results further confirm that elemental Ti serves as the primary source of the photothermal effect [62]. As such, we speculated that the photothermal effect may play an important role in the CH_3_OH decomposition reaction. To further validate this point, a temperature-controlled experiment was carried out. As depicted in Figs. 3a and S28–S30, under normal illumination conditions (maintained at 333 K), the yield rates of H_2_, HCHO, and CO were 0.511, 0.485, and 0.017 mmol h^−1^ over Cu–TiO_2_/Ti, respectively. However, when the reaction was performed under irradiation while maintaining the reaction temperature at room temperature using a water-cooling system (maintained at 293 K), the formation of phonons contributed from the photothermal effect was efficiently suppressed. In this case, the yield rates of H_2_, HCHO, and CO reduced to 0.117, 0.082, and 0.027 mmol h^−1^, respectively. It is noteworthy that although HCHO remained the main oxidation product, the proportion of CO product obviously increased from 3.38% to 24.99%. This result is quite counterintuitive, as overoxidation of HCHO to CO typically favors higher temperatures. It means that the photothermal effect not only influenced the activity by providing extra thermal energy, but also inhibited the overoxidation of HCHO to CO. Moreover, under dark conditions with only external heating applied, the H_2_ production rate of the Cu–TiO_2_/Ti catalyst at 333 K was only 0.019 mmol h⁻^1^, confirming that photon excitation is indispensable for initiating the photocatalytic reaction (Fig. S31). Furthermore, the respective contributions of the photothermal effect, photochemical effect, and photochemical-thermo synergistic effect to the H_2_ evolution process were quantitatively analyzed (Table S11). It was found that the contribution of the pure photothermal effect was only approximately 3.7%, while the pure photochemical effect contributed 22.9%, and the synergistic contribution of the photochemical-thermo synergistic effect reached 73.4%. These results indicate that the photochemical effect is the key factor for initiating the H_2_ evolution reaction, whereas the photochemical-thermo synergistic effect can substantially promote the H_2_ evolution process, serving as a crucial driving force for the rapid photothermal catalytic methanol dehydrogenation reaction [63–66].

To further investigate the vital role of the photothermal effect in photocatalytic behavior, we performed in situ diffuse reflectance infrared spectroscopy (DRIFTS) of Cu–TiO_2_/Ti catalysts (Fig. S32). To simulate reaction conditions, the phonon effect arising from the photothermal process was also restrained by controlling the temperature with a condensate system. As shown in Fig. 3b, the band at 1821 cm^–1^ originated from the stretching vibration mode of the C=O bond in the CH_2_O* species. Moreover, the CO* species was observed at 2075 cm^–1^ [67–69]. Apparently, when the condensate water was introduced, the intensity of the CO* species enhanced significantly, while the CH_2_O* signal decreased (Fig. 3b). As such, suppressing phonons would facilitate the transformation from HCHO to CO, which was consistent with the production selectivity trend.

The above findings were further verified by in situ synchrotron radiation photoelectron spectroscopy (SRPES) tests (Figs. S32 and S33). During the experiments, the Cu–TiO_2_/Ti sample was treated with 1 bar of Ar, which bubbled in CH_3_OH, followed by light irradiation for varying durations. Meanwhile, the phonon effect was controlled via changing the temperature with or without the introduction of liquid nitrogen into the analysis chamber (Fig. S33). Under standard reaction conditions, the C 1*s* spectra exhibited three typical peaks at 285.4, 286.9, and 288.4 eV, corresponding to CH_3_OH*, CH_3_O*, and CH_2_O* species, respectively (Fig. 3c) [70, 71]. It suggested that the transformation of adsorbed CH_3_OH proceeds via a stepwise formation of CH_3_O* and CH_2_O*. In addition, a weak peak assigned to CO_x_* species was observed at 290.5 eV, indicating that few CH_2_O* were converted into CO_x_* species. Quantitative analysis of the time-dependent C 1*s* spectra (Table S12) shows that under light irradiation without cooling (temperature maintained at 333 K), the CH_2_O* signal increases from 24.98 at 5 min to 50.02% at 30 min, while the COₓ* signal remains low (≤ 10.41%). When the test was carried out with light irradiation and with liquid nitrogen cooling, the reaction temperature was reduced, and phonons were significantly suppressed. In this case, both CH_3_OH* and CH_3_O* were also observed. However, different from the above, the increase in CH_2_O* slowed down while the proportion of CO_x_* species increased. Specifically, under the phonon-suppressed condition (293 K), the CH_2_O* signal increases only from 6.98 to 13.45% over 30 min, whereas the COₓ* signal rises from 4.31% to 17.55%. This implied that, in the absence of phonons, more CH_2_O* species further underwent oxidative dehydrogenation to form CO_x_* species. Thus, we speculated that phonons may facilitate the desorption of generated HCHO to prevent overoxidation to CO.

Molecular dynamics (MD) simulations were further employed to investigate the desorption process of HCHO over the Cu–TiO_2_(110) model (Fig. 3d). As shown in Fig. 3e, the collective variable (CV) value presented the sum of the z-axis coordinates of the four atoms in the HCHO molecule. The increase in CV value corresponded to the gradual desorption process of HCHO from the model surface. As for the adsorbed HCHO species, the energy change of the CV value from 2.6 to 3.2 was determined as 0.58 eV at 293 K, while at 333 K, the energy change decreased to 0.52 eV. Consequently, the MD simulation results directly revealed that hot phonons could facilitate the desorption of HCHO species by lowering the energy barrier (Fig. S34) [72, 73]. Based on the above results, we can conclude that phonons from the photothermal effect accelerated the photocatalytic conversion of CH_3_OH into HCHO and H_2_, meanwhile promoted the desorption of generated HCHO to avoid the over-oxidation to CO product.

We proposed a reaction pathway of CH_3_OH decomposition over Cu–TiO_2_/Ti. Upon illumination, TiO_2_ underwent a photochemical process to generate holes and electrons, which accumulated at the Ti sites surrounding the Cu atoms and at the Cu sites due to the construction of an efficient electron transfer channel from the Ti to Cu atoms, respectively. CH_3_OH was initially adsorbed over the Ti sites and was subsequently converted through a stepwise formation of CH_3_O* and CH_2_O* via reaction with photogenerated holes (Fig. S35). Simultaneously, the photogenerated electrons that accumulated at Cu sites led to the formation of Cu^*δ*+^ (1 < *δ* < 2) sites, which in turn transform two released H atoms into H_2_. The CH_2_O* species then desorbed from the catalyst surface, finally generating HCHO. During the reaction, the photochemical effect from TiO_2_ acted as the primary driving force, not only providing photogenerated carriers for the stepwise reactions of CH_3_OH to H_2_ and HCHO, but also lowering the reaction barrier for H_2_ production over Cu^*δ*+^ (1 < *δ* < 2) sites by photogenerated electrons. In this context, the photothermal effect from the elemental Ti enhanced catalytic activity by accelerating the reaction rate. Besides, the suppression of CO formation was attributed to phonon-mediated desorption of HCHO, which prevented its further overoxidation.

**Fig. 4:**
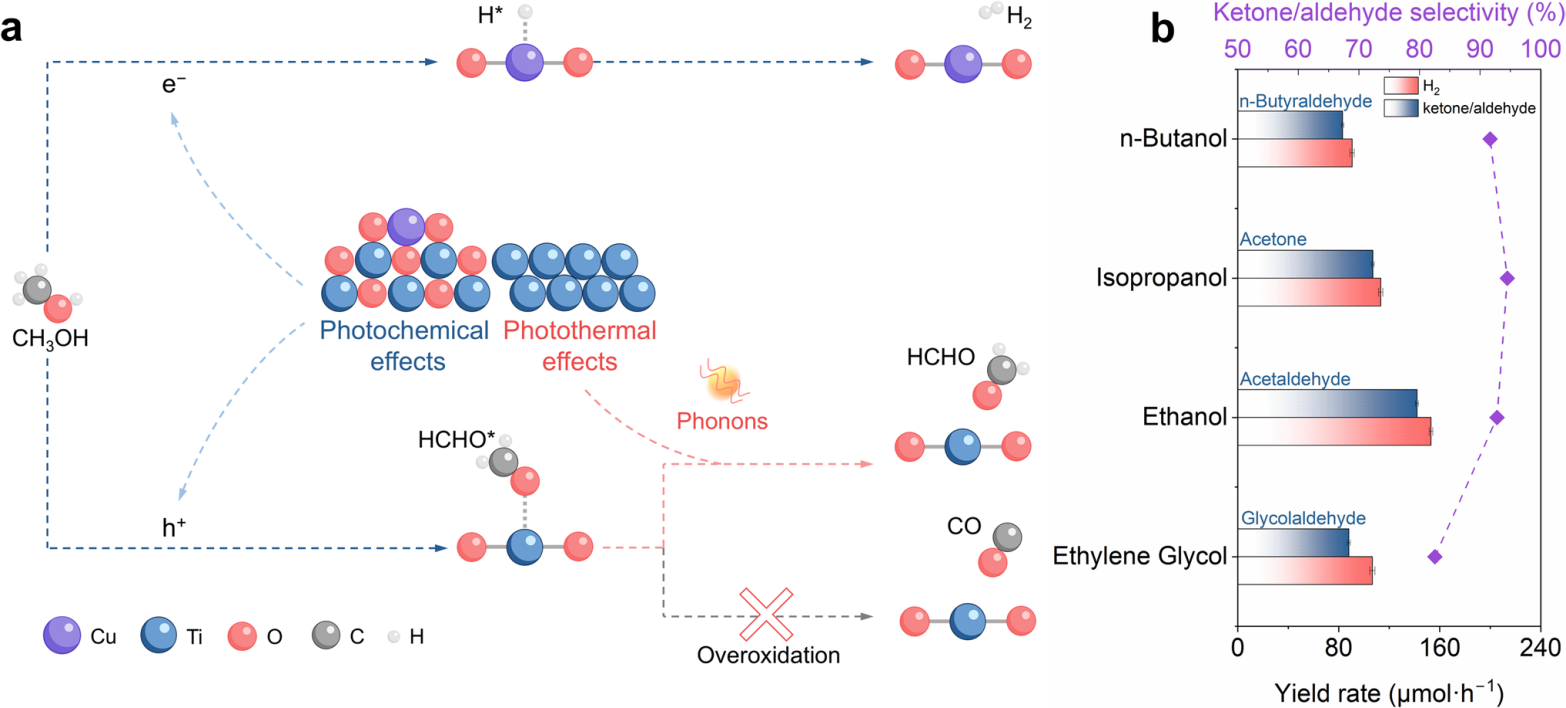
**a** Schematic diagram of the reaction mechanism over Cu-TiO_2_/Ti. **b** Expansion experiments of productions from different alcohols over Cu-TiO_2_/Ti (Light intensity: 500 mW cm^−2^)

In addition to methanol, we also expanded our investigations using other alcohols as reactants, such as ethanol, ethylene glycol, isopropanol, and n-butanol. As depicted in Fig. 4b, as for ethanol system, the H_2_ production rate reached 153 μmol h^−1^ and acetaldehyde yield was 142 μmol h^−1^ with a selectivity of 92.8%. When it comes to ethylene glycol, we achieved an H_2_ evolution rate of 106 μmol h^−1^ alongside glycolaldehyde product formation at 88 μmol h^−1^, with a selectivity of 83.0%. For isopropanol, the yield rates of H_2_ and acetone were 113 and 107 μmol h^−1^, and the selectivity of acetone was calculated as 94.7%. In addition, the yield rates of H_2_ and n-butyraldehyde in n-butanol system were 91 and 83 μmol h^−1^ (91.2% selectivity). These results indicate that the Cu–TiO_2_/Ti photocatalyst also exhibits high H_2_ evolution and high selectivity toward ketone/aldehyde products during the dehydrogenation of other alcohols, demonstrating broad universality.

## Conclusion

In conclusion, we elucidated the synergetic effects of photochemical and photothermal distribution in driving efficient photocatalytic reactions, while recognizing that these two effects are intrinsically coupled under operating conditions. The photochemical process served as the primary driving force, supplying photogenerated holes for the stepwise dehydrogenation of CH_3_OH to HCHO and photogenerated electrons for accelerating H_2_ production at Cu^*δ*+^ (1 < *δ* < 2) sites. The accumulation of photogenerated electrons at Cu single atoms facilitated efficient charge separation, as supported by in situ RAS, in situ XAFS, and DFT calculations. Concurrently, the hot phonons from the photothermal effect suppressed the overoxidation to CO by reducing the energy barrier for HCHO desorption. Benefiting from this, an impressive H_2_ production rate of 0.511 mmol h^−1^ with 96.6% of HCHO selectivity was obtained over Cu–TiO_2_/Ti from CH_3_OH. Furthermore, Cu–TiO_2_/Ti also displayed outstanding performance in the expansion experiments using other alcohols as reactants. This work not only highlights the importance of coupling photochemical and photothermal processes for enhanced photocatalytic performance but also offers mechanistic insights for the rational design of efficient solar-driven systems for alcohol decomposition.

## Supplementary Information

Below is the link to the electronic supplementary material.

Supplementary file1 (DOC 7444 KB)

## References

[1] C. Song, Z. Wang, Z. Yin, D. Xiao, D. Ma, Principles and applications of photothermal catalysis. Chem. Catal. **2**(1), 52–83 (2022). 10.1016/j.checat.2021.10.005

[2] H. Song, K. Sun, H. Huang, S. Ning, S. Wang et al., Integrating photochemical and photothermal effects for selective oxidative coupling of methane into C^2+^ hydrocarbons with multiple active sites. Nat. Commun. **16**, 2831 (2025). 10.1038/s41467-025-58101-0 40121218 10.1038/s41467-025-58101-0 PMC11929825

[3] R. Li, Y. Li, Z. Li, S. Ouyang, H. Yuan et al., Unleashing the full potential of photo-driven CO hydrogenation to light olefins over carbon-coated CoMn-based catalysts. Adv. Mater. **35**(44), e2307217 (2023). 10.1002/adma.202307217 37704217 10.1002/adma.202307217

[4] N. Sun, X. Liu, C. Tian, Q. Xu, Y. Xuan, Selective plasmonic C─H bond editing for low-temperature light-driven greenhouse gas upgrading. Adv. Energy Mater. **15**(5), 2404005 (2025). 10.1002/aenm.202404005

[5] Y. Yuan, J. Zhou, A. Bayles, H. Robatjazi, P. Nordlander et al., Steam methane reforming using a regenerable antenna–reactor plasmonic photocatalyst. Nat. Catal. **7**(12), 1339–1349 (2024). 10.1038/s41929-024-01248-8

[6] C. Xu, Q. Tang, W. Tu, L. Wang, Photon and phonon powered photothermal catalysis. Energy Environ. Sci. **17**(13), 4461–4480 (2024). 10.1039/d4ee00783b

[7] D. He, G. Huang, Z. Zhou, Q. Hu, J. Ding et al., Recent progress for designing of catalysts for photothermal conversion of plastic wastes. Adv. Funct. Mater. **36**(6), 2419801 (2026). 10.1002/adfm.202419801

[8] C. Xing, C. Mao, S. Wang, Y. Zhou, L. Wu et al., Ambient solar thermal catalysis for polyolefin upcycling using copper encapsulated in silicon nanosheets and chloroaluminate ionic liquid. Nat. Catal. **8**(6), 556–568 (2025). 10.1038/s41929-025-01349-y

[9] J.-H. Mei, Y.-R. Zeng, Y.-N. Gong, W.-J. Shi, D.-C. Zhong et al., π–π stacking as electron-transfer channels in hydrogen-bonded organic frameworks for boosting photocatalysis. Angew. Chem. Int. Ed. **64**(29), e202507332 (2025). 10.1002/anie.202507332 10.1002/anie.202507332 40359071

[10] J. Xu, M. Chong, W. Li, E. Zhu, H. Jin, L. Liu, Y. Ren, Y. Zhu, Guiding electron transfer for selective C_2_H_6_ formation from CO_2_ photoreduction via built-in electric fields. Chem **39**, 104856 (2025). 10.1016/j.chempr.2024.08.018

[11] B.-B. Luan, X. Chu, Y. Wang, X. Qiao, Y. Jiang et al., Construction of COF/COF organic S-scheme heterostructure for enhanced overall water splitting. Adv. Mater. **36**(49), 2412653 (2024). 10.1002/adma.202412653 10.1002/adma.202412653 39422373

[12] M. Dan, S. Yu, W. Lin, M. Abdellah, Z. Guo et al., Balancing the charge separation and surface reaction dynamics in twin-interface photocatalysts for solar-to-hydrogen production. Adv. Mater. **37**(4), 2415138 (2025). 10.1002/adma.202415138 10.1002/adma.202415138 39558773

[13] Y. Li, H. Zhou, S. Cai, D. Prabhakaran, W. Niu et al., Electrolyte-assisted polarization leading to enhanced charge separation and solar-to-hydrogen conversion efficiency of seawater splitting. Nat. Catal. **7**(1), 77–88 (2024). 10.1038/s41929-023-01069-1

[14] C. Choi, F. Zhao, J.L. Hart, Y. Gao, F. Menges et al., Synergizing electron and heat flows in photocatalyst for direct conversion of captured CO_2_. Angew. Chem. Int. Ed. **62**(23), e202302152 (2023). 10.1002/anie.202302152 10.1002/anie.202302152 36972027

[15] Z. Ma, S. Zhan, Y. Zhang, A. Kuklin, Y. Chen et al., An electron transfer mediated mechanism for efficient photoreforming of waste plastics using a Ni_3_S_4_/ZnCdS heterojunction. Adv. Mater. **37**(14), 2416581 (2025). 10.1002/adma.202416581 39989159 10.1002/adma.202416581 PMC11983256

[16] J. Meng, K. Wang, Y. Wang, J. Ma, C. Ban et al., Bismuth clusters pinned on TiO_2_ porous nanowires boosting charge transfer for CO_2_ photoreduction to CH_4_. Nano Res. **17**(3), 1190–1198 (2024). 10.1007/s12274-023-5990-6

[17] H. Zhang, J. Zhou, X. Li, Y. Han, N. Zhang et al., Elucidating Raman auger behavior in Cu, Cu_2_O and CuO by resonant auger electron spectroscopy to interpret X-ray absorption spectroscopy. Small Methods **9**(10), 2402027 (2025). 10.1002/smtd.202402027 10.1002/smtd.202402027 40181616

[18] C.-Y. Tang, K. Leung, R.T. Haasch, S.J. Dillon, LiMn_2_O_4_ surface chemistry evolution during cycling revealed by *in situ* auger electron spectroscopy and X-ray photoelectron spectroscopy. ACS Appl. Mater. Interfaces **9**(39), 33968–33978 (2017). 10.1021/acsami.7b10442 28901735 10.1021/acsami.7b10442

[19] Y. Li, B. Liu, D. Yuan, H. Wang, Q. Wu et al., High-purity carbon monoxide production *via* photothermal formic acid decomposition over fluorite ZrO_2_. Nat. Catal. **7**(12), 1350–1358 (2024). 10.1038/s41929-024-01249-7

[20] Y. Zhang, B. Sun, C. Cai, T. Wang, Y. Gao et al., Photothermocatalytic wet reforming of waste plastics to syngas. J. Am. Chem. Soc. **147**(11), 9879–9890 (2025). 10.1021/jacs.5c00620 40019224 10.1021/jacs.5c00620

[21] Y. Li, X. Bai, D. Yuan, F. Zhang, B. Li et al., General heterostructure strategy of photothermal materials for scalable solar-heating hydrogen production without the consumption of artificial energy. Nat. Commun. **13**, 776 (2022). 10.1038/s41467-022-28364-y 35140217 10.1038/s41467-022-28364-y PMC8828830

[22] S. Liu, X. Wang, Y. Chen, Y. Li, Y. Wei et al., Efficient thermal management with selective metamaterial absorber for boosting photothermal CO_2_ hydrogenation under sunlight. Adv. Mater. **36**(21), 2311957 (2024). 10.1002/adma.202311957 10.1002/adma.202311957 38324747

[23] S. Fregert, I. Dahlquist, B. Gruvberger, A simple method for the detection of formaldehyde. Contact Dermat. **10**(3), 132–134 (1984). 10.1111/j.1600-0536.1984.tb00017.x 10.1111/j.1600-0536.1984.tb00017.x 6713848

[24] R. Zhang, H. Xu, Z. Huang, J. Zhang, L. Liu et al., Synergistic redox dual-site strategy to boost photosynthesis of hydrogen peroxide. Adv. Funct. Mater. **35**(15), 2420504 (2025). 10.1002/adfm.202420504

[25] G. Xing, M. Tong, P. Yu, L. Wang, G. Zhang et al., Reconstruction of highly dense Cu-N(4) active sites in electrocatalytic oxygen reduction characterized by operando synchrotron radiation. Angew. Chem. Int. Ed. **61**(40), e202211098 (2022). 10.1002/anie.202211098 10.1002/anie.202211098 35993239

[26] J. Gu, M. Jian, L. Huang, Z. Sun, A. Li et al., Synergizing metal–support interactions and spatial confinement boosts dynamics of atomic nickel for hydrogenations. Nat. Nanotechnol. **16**(10), 1141–1149 (2021). 10.1038/s41565-021-00951-y 34312515 10.1038/s41565-021-00951-y

[27] S. Chen, H. Wang, Z. Kang, S. Jin, X. Zhang et al., Oxygen vacancy associated single-electron transfer for photofixation of CO_2_ to long-chain chemicals. Nat. Commun. **10**(1), 788 (2019). 10.1038/s41467-019-08697-x 30770824 10.1038/s41467-019-08697-x PMC6377667

[28] G. Kresse, J. Furthmüller, Efficiency of ab-initio total energy calculations for metals and semiconductors using a plane-wave basis set. Comput. Mater. Sci. **6**(1), 15–50 (1996). 10.1016/0927-0256(96)00008-0 10.1103/physrevb.54.11169 9984901

[29] G. Kresse, D. Joubert, From ultrasoft pseudopotentials to the projector augmented-wave method. Phys. Rev. B **59**(3), 1758–1775 (1999). 10.1103/physrevb.59.1758

[30] A. Migani, L. Blancafort, Excitonic interfacial proton-coupled electron transfer mechanism in the photocatalytic oxidation of methanol to formaldehyde on TiO_2_(110). J. Am. Chem. Soc. **138**(49), 16165–16173 (2016). 10.1021/jacs.6b11067 27960348 10.1021/jacs.6b11067

[31] B.J. Morgan, G.W. Watson, A DFT+U description of oxygen vacancies at the TiO_2_ rutile (110) surface. Surf. Sci. **601**(21), 5034–5041 (2007). 10.1016/j.susc.2007.08.025

[32] S. Grimme, J. Antony, S. Ehrlich, H. Krieg, A consistent and accurate *ab initio* parametrization of density functional dispersion correction (DFT-D) for the 94 elements H-Pu. J. Chem. Phys. **132**(15), 154104 (2010). 10.1063/1.3382344 20423165 10.1063/1.3382344

[33] W. Yuan, B. Chen, Z.-K. Han, R. You, Y. Jiang et al., Direct *in-situ* insights into the asymmetric surface reconstruction of rutile TiO_2_ (110). Nat. Commun. **15**, 1616 (2024). 10.1038/s41467-024-46011-6 38388567 10.1038/s41467-024-46011-6 PMC10883989

[34] M.C. Biesinger, B.P. Payne, B.R. Hart, A.P. Grosvenor, N.S. McIntryre et al., Quantitative chemical state XPS analysis of first row transition metals, oxides and hydroxides. J. Phys. Conf. Ser. **100**(1), 012025 (2008). 10.1088/1742-6596/100/1/012025

[35] D. Lützenkirchen-Hecht, M. Wagemaker, P. Keil, A.A. van Well, R. Frahm, *Ex situ* reflection mode EXAFS at the Ti K-edge of lithium intercalated TiO_2_ rutile. Surf. Sci. **538**(1–2), 10–22 (2003). 10.1016/S0039-6028(03)00722-2

[36] A. Gaur, B.D. Shrivastava, S.K. Joshi, Copper K-edge XANES of Cu(I) and Cu(II) oxide mixtures. J. Phys. Conf. Ser. **190**, 012084 (2009). 10.1088/1742-6596/190/1/012084

[37] Y. Shen, C. Ren, L. Zheng, X. Xu, R. Long et al., Room-temperature photosynthesis of propane from CO_2_ with Cu single atoms on vacancy-rich TiO_2_. Nat. Commun. **14**, 1117 (2023). 10.1038/s41467-023-36778-5 36849519 10.1038/s41467-023-36778-5 PMC9970977

[38] J. Ding, L. Liu, J. Zhang, Y. Liu, H. Xu et al., Unraveling dynamic structural evolution of single atom catalyst *via in situ* surface-enhanced infrared absorption spectroscopy. J. Am. Chem. Soc. **147**(11), 9601–9609 (2025). 10.1021/jacs.4c17565 40054996 10.1021/jacs.4c17565

[39] S. Rej, L. Mascaretti, E.Y. Santiago, O. Tomanec, Š Kment et al., Determining plasmonic hot electrons and photothermal effects during H_2_ evolution with TiN–Pt nanohybrids. ACS Catal. **10**(9), 5261–5271 (2020). 10.1021/acscatal.0c00343

[40] B.-H. Lee, S. Park, M. Kim, A.K. Sinha, S.C. Lee et al., Reversible and cooperative photoactivation of single-atom Cu/TiO_2_ photocatalysts. Nat. Mater. **18**(6), 620–626 (2019). 10.1038/s41563-019-0344-1 31011217 10.1038/s41563-019-0344-1

[41] L. Liu, Y. Sun, Z. Ma, Q. Liu, R. Zhang et al., Vacancy-induced symmetry breaking in titanium dioxide boosts the photocatalytic hydrogen production from methanol aqueous solution. Nano Lett. (2024). 10.1021/acs.nanolett.4c03696 10.1021/acs.nanolett.4c03696 39353098

[42] Y.-J. Yuan, Z.-J. Ye, H.-W. Lu, B. Hu, Y.-H. Li et al., Constructing anatase TiO_2_ nanosheets with exposed (001) facets/layered MoS_2_ two-dimensional nanojunctions for enhanced solar hydrogen generation. ACS Catal. **6**(2), 532–541 (2016). 10.1021/acscatal.5b02036

[43] T.A. Kandiel, F. Tyrsted, K.R. Petersen, N.P. Rønnau, O.A. Ljunggren, S.E. Bjørheim, U.B. Wolff, Photonic efficiency and mechanism of photocatalytic hydrogen production over TiO_2_: effect of particle size and surface treatment. Catal. Today **164**, 25 (2011). 10.1016/j.cattod.2010.08.012

[44] W.-T. Chen, A. Chan, D. Sun-Waterhouse, J. Llorca, H. Idriss et al., Performance comparison of Ni/TiO_2_ and Au/TiO_2_ photocatalysts for H_2_ production in different alcohol-water mixtures. J. Catal. **367**, 27–42 (2018). 10.1016/j.jcat.2018.08.015

[45] G. Ou, Y. Xu, B. Wen, R. Lin, B. Ge et al., Tuning defects in oxides at room temperature by lithium reduction. Nat. Commun. **9**, 1302 (2018). 10.1038/s41467-018-03765-0 29615620 10.1038/s41467-018-03765-0 PMC5882908

[46] J. Luo, C. Zhu, J. Li, J. Jin, N.E. Soland et al., Photocatalytic methanol dehydrogenation with switchable selectivity. J. Am. Chem. Soc. **147**(4), 3428–3437 (2025). 10.1021/jacs.4c14413 39804253 10.1021/jacs.4c14413

[47] G. Jeantelot, M. Qureshi, M. Harb, S. Ould-Chikh, D.H. Anjum et al., TiO_2_-supported Pt single atoms by surface organometallic chemistry for photocatalytic hydrogen evolution. Phys. Chem. Chem. Phys. **21**(44), 24429–24440 (2019). 10.1039/C9CP04470A 31674630 10.1039/c9cp04470a

[48] M. Xiao, A. Baktash, M. Lyu, G. Zhao, Y. Jin et al., Unveiling the role of water in heterogeneous photocatalysis of methanol conversion for efficient hydrogen production. Angew. Chem. Int. Ed. **63**(21), e202402004 (2024). 10.1002/anie.202402004 10.1002/anie.202402004 38531783

[49] C.G. Silva, M.J. Sampaio, R.R.N. Marques, L.A. Ferreira, P.B. Tavares et al., Photocatalytic production of hydrogen from methanol and TiO_2_/carbon nanotube composite materials. Int. J. Hydrogen Energy **40**, 1015–1025 (2015). 10.1016/j.apcatb.2014.10.032

[50] M. Zhou, S. Xue, Q. Feng, X. Liang, W. Wu et al., Carbon layers on Pt/TiO_2_ induced dramatic promotion of photocatalytic H_2_ production: a combined experimental and computation study. Mater. Today Energy **34**, 101294 (2023). 10.1016/j.mtener.2023.101294

[51] B. Xia, B. He, J. Zhang, L. Li, Y. Zhang et al., TiO_2_/FePS_3_ S-scheme heterojunction for greatly raised photocatalytic hydrogen evolution. Adv. Energy Mater. **12**(46), 2201449 (2022). 10.1002/aenm.202201449

[52] Y. Liu, B. Zhang, L. Luo, X. Chen, Z. Wang et al., TiO_2_/Cu_2_O core/ultrathin shell nanorods as efficient and stable photocatalysts for water reduction. Angew. Chem. Int. Ed. **54**(50), 15260–15265 (2015). 10.1002/anie.201509115 10.1002/anie.201509115 26555557

[53] W. Wang, S. Zhu, Y. Cao, Y. Tao, X. Li et al., Edge-enriched ultrathin MoS_2_ embedded yolk-shell TiO_2_ with boosted charge transfer for superior photocatalytic H_2_ evolution. Adv. Funct. Mater. **29**(36), 1901958 (2019). 10.1002/adfm.201901958

[54] A. Kotani, K.O. Kvashnina, S.M. Butorin, P. Glatzel, Spectator and participator processes in the resonant photon-in and photon-out spectra at the Ce L3 edge of CeO_2_. Eur. Phys. J. B. **85**(8), 257 (2012). 10.1140/epjb/e2012-30079-1

[55] F. Trinter, M.S. Schöffler, H.-K. Kim, F.P. Sturm, K. Cole et al., Resonant Auger decay driving intermolecular Coulombic decay in molecular dimers. Nature **505**(7485), 664–666 (2014). 10.1038/nature12927 24362568 10.1038/nature12927

[56] Y. Tanaka, M. Karppinen, J.M. Lee, R.S. Liu, J.M. Chen et al., Systematic CuL2, 3-edge and OK-edge XANES spectroscopy study on the infinite-layer superconductor system, (Sr, La)CuO_2_. Solid State Commun. **147**(9–10), 370–373 (2008). 10.1016/j.ssc.2008.06.024

[57] N. Pauly, S. Tougaard, F. Yubero, LMM Auger primary excitation spectra of copper. Surf. Sci. **630**, 294–299 (2014). 10.1016/j.susc.2014.08.029

[58] Q. Ma, D. Li, F. Ren, W. Gao, R. Song et al., Photoelectrocatalytic degradation mechanism of fluorinated pollutants using a bilayer WO_3_ photoanode: synergistic role of holes and hydroxyl radicals. Angew. Chem. Int. Ed. **64**(25), e202506322 (2025). 10.1002/anie.202506322 10.1002/anie.202506322 40178180

[59] Q. Chen, J. Huang, D. Chu, L. Cao, X. Li et al., Accelerated photogenerated charge separation driven synergistically by the interfacial electric field and work function in Z-scheme Zn-Ni_2_P/G-C_3_N_4_ for efficient photocatalytic hydrogen evolution. Exploration **5**(5), 20240189 (2025). 10.1002/EXP.20240189 41163800 10.1002/EXP.20240189 PMC12561414

[60] L. Lv, Y. Liu, X. Li, Y. Huang, T. Li et al., Synergistic engineering of zinc vacancies and Er-doping in ZnIn2S4 nanosheets for enhanced CO_2_ photoreduction *via* optimized charge dynamics. Carbon Neutralization **4**(4), e70021 (2025). 10.1002/cnl2.70021

[61] C. Ban, Y. Wang, Y. Feng, Z. Zhu, Y. Duan et al., Photochromic single atom Ag/TiO_2_ catalysts for selective CO_2_ reduction to CH_4_. Energy Environ. Sci. **17**(2), 518–530 (2024). 10.1039/D3EE02800C

[62] C. Zhan, B.-W. Liu, Y.-F. Huang, S. Hu, B. Ren et al., Disentangling charge carrier from photothermal effects in plasmonic metal nanostructures. Nat. Commun. **10**(1), 2671 (2019). 10.1038/s41467-019-10771-3 31209216 10.1038/s41467-019-10771-3 PMC6572789

[63] C. Liu, S. Tang, R. Song, Y.-F. Xu, G.A. Ozin et al., Micro/nanoscale thermometry in photothermal catalysis. Joule **9**(8), 102052 (2025). 10.1016/j.joule.2025.102052

[64] Y.-F. Xu, A.A. Tountas, R. Song, J. Ye, J.-H. Wei et al., Equilibrium photo-thermodynamics enables a sustainable methanol synthesis. Joule **7**(4), 738–752 (2023). 10.1016/j.joule.2023.03.014

[65] X. Bian, Y. Zhao, G.I.N. Waterhouse, Y. Miao, C. Zhou et al., Quantifying the contribution of hot electrons in photothermal catalysis: a case study of ammonia synthesis over carbon-supported Ru catalyst. Angew. Chem. Int. Ed. **62**(25), e202304452 (2023). 10.1002/anie.202304452 10.1002/anie.202304452 37083180

[66] X. Bian, Y. Zhao, C. Zhou, T. Zhang, Minimizing temperature bias through reliable temperature determination in gas-solid photothermal catalytic reactions. Angew. Chem. Int. Ed. **62**(24), e202219340 (2023). 10.1002/anie.202219340 10.1002/anie.202219340 37060210

[67] D. Li, Y. Li, X. Liu, Y. Guo, C.-W. Pao et al., NiAl_2_O_4_ spinel supported Pt catalyst: high performance and origin in aqueous-phase reforming of methanol. ACS Catal. **9**(10), 9671–9682 (2019). 10.1021/acscatal.9b02243

[68] J. Li, B. Sheng, Y. Chen, J. Yang, T. Ma et al., Nickel–iron bimetal as a cost-effective cocatalyst for light-driven hydrogen release from methanol and water. ACS Catal. **13**(15), 10153–10160 (2023). 10.1021/acscatal.3c02024

[69] Q. Wang, Y. Wan, Q. Liu, Y. Zhang, Z. Ma et al., A multi-site Ru-Cu/CeO_2_ photocatalyst for boosting C-N coupling toward urea synthesis. Sci. Bull. **70**(7), 1118–1125 (2025). 10.1016/j.scib.2025.01.059 10.1016/j.scib.2025.01.059 39947988

[70] A. Schaefer, W. Cartas, R. Rai, M. Shipilin, L.R. Merte et al., Methanol adsorption and oxidation on reduced and oxidized TbO_*x*_(111) surfaces. J. Phys. Chem. C **120**(50), 28617–28629 (2016). 10.1021/acs.jpcc.6b09908

[71] V. Matolín, J. Libra, M. Škoda, N. Tsud, K.C. Prince et al., Methanol adsorption on a CeO_2_(111)/Cu(111) thin film model catalyst. Surf. Sci. **603**(8), 1087–1092 (2009). 10.1016/j.susc.2009.02.010

[72] X. Bai, X. Zhao, Y. Zhang, C. Ling, Y. Zhou et al., Dynamic stability of copper single-atom catalysts under working conditions. J. Am. Chem. Soc. **144**(37), 17140–17148 (2022). 10.1021/jacs.2c07178 36089737 10.1021/jacs.2c07178

[73] H. Cao, Z. Zhang, J.-W. Chen, Y.-G. Wang, Potential-dependent free energy relationship in interpreting the electrochemical performance of CO_2_ reduction on single atom catalysts. ACS Catal. **12**(11), 6606–6617 (2022). 10.1021/acscatal.2c01470

