# Supplementary material for: Elucidating the Respective Roles of Photochemical and Photothermal Effects in Photocatalytic Methanol Decomposition
Source: Nanomicro Lett. 2026 Jul 27;19:2. doi: 10.1007/s40820-026-02305-6 (PMC13407809; doi:10.1007/s40820-026-02305-6)
Supplement: Supplementary file 1 — Supplementary file1 (DOC 7444 KB) [file 40820_2026_2305_MOESM1_ESM.doc]

Supporting Information for

**Elucidating the Respective Roles of Photochemical and Photothermal Effects in Photocatalytic Methanol Decomposition**

Qichen Liu^1, 2†^, Yida Zhang^3†^, Limin Liu^1†^, Zixiang Huang^1, 4^, Jiawei Zheng^1, 5^, Shiqin Jian^6^, Haibin Pan^1^, Chi Cao^1^, Hongliang Li^4^, Qing Yang^2^, *, Yu Bai^7, *^, Xusheng Zheng^1^, *

^1^ National Synchrotron Radiation Laboratory (NSRL), University of Science and Technology of China, Hefei, Anhui 230029, P. R. China

^2^ College of Chemistry and Materials Science, University of Science and Technology of China, Hefei, Anhui 230026, P. R. China

^3^ College of Chemical Engineering, Inner Mongolia University of Technology, Hohhot, Inner Mongolia 010051, P. R. China

^4^ Hefei National Research Center for Physical Sciences at the Microscale, University of Science and Technology of China, Hefei, Anhui 230026, P. R. China

^5^ School of Nuclear Science and Technology, Southwest University of Science and Technology, Mianyang, Sichuan 621010, P. R. China

^6^ School of Physics and Mechanics, Wuhan University of Technology, Wuhan, Hubei 430070, P. R. China

^7^ Experimental Center for Engineering and Materials Science, University of Science and Technology of China, Hefei, Anhui 230027, P. R. China

^†^Qichen Liu, Yida Zhang, and Limin Liu contributed equally to this paper.

* Corresponding authors. E-mail: [qyoung@ustc.edu.cn](mailto:qyoung@ustc.edu.cn) (Qing Yang), [baiyu@ustc.edu.cn](mailto:baiyu@ustc.edu.cn) (Yu Bai); [zxs@ustc.edu.cn](mailto:zxs@ustc.edu.cn) (Xusheng Zheng)

**S1 Supplementary Figures and Tables**


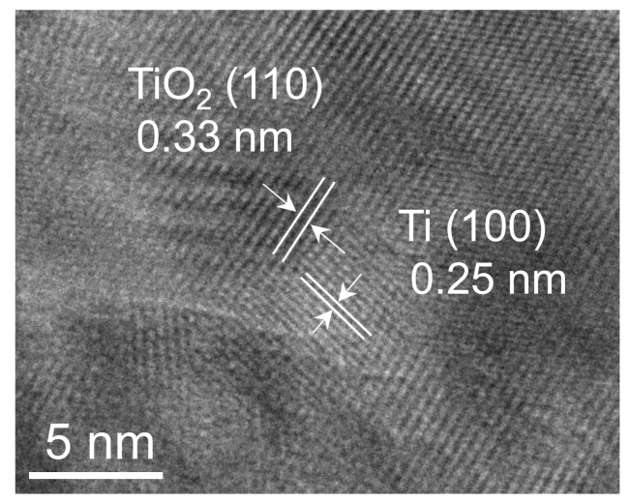


**Fig. S1** HRTEM image of Cu-TiO_2_/Ti sample.


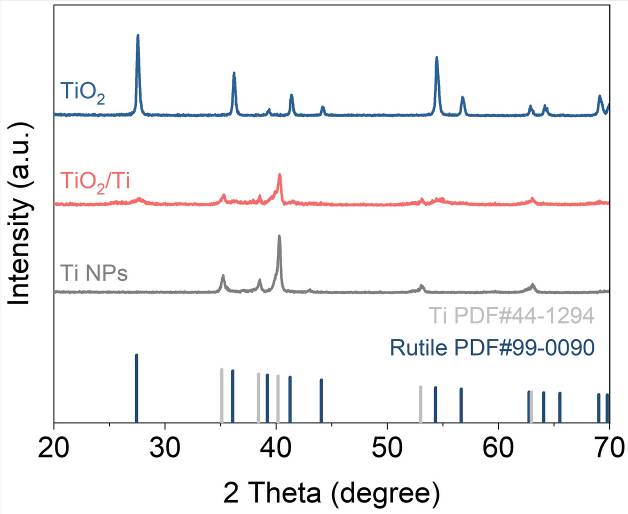


**Fig. S2** XRD patterns of Ti NPs, TiO_2_/Ti, and TiO_2_ samples, respectively.


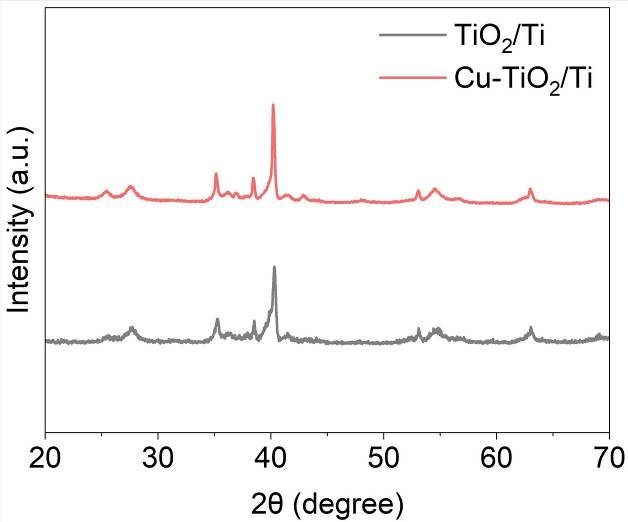


**Fig. S3** XRD patterns of TiO_2_/Ti and Cu-TiO_2_/Ti samples.


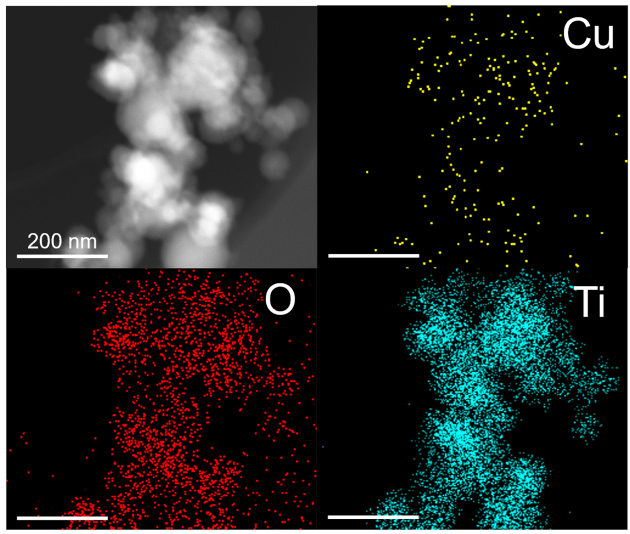


**Fig. S4** Elemental mapping images of Cu-TiO_2_/Ti sample.


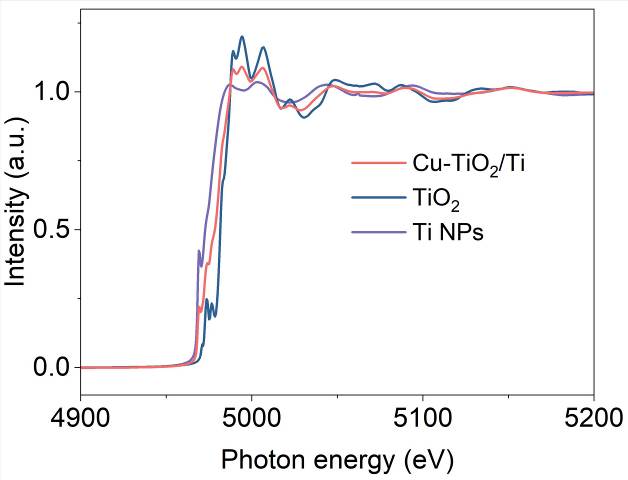


**Fig. S5** Ti K-edge XANES spectra for Ti NPs, TiO_2_, and Cu-TiO_2_/Ti samples.


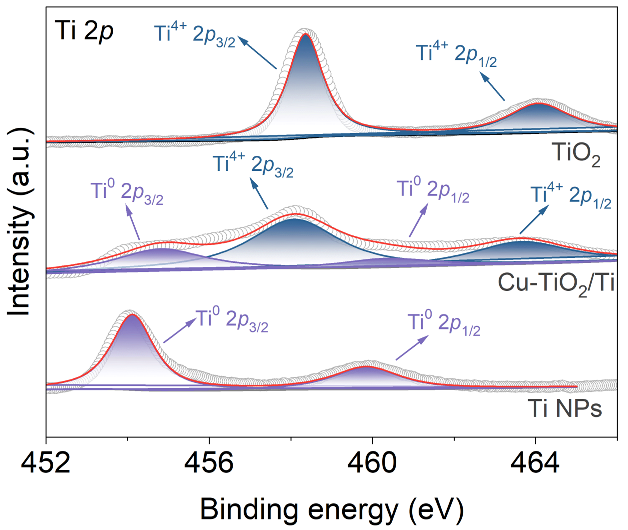


**Fig. S6** Ti *2p* spectra for Ti NPs, Cu-TiO_2_/Ti, and TiO_2_ samples.


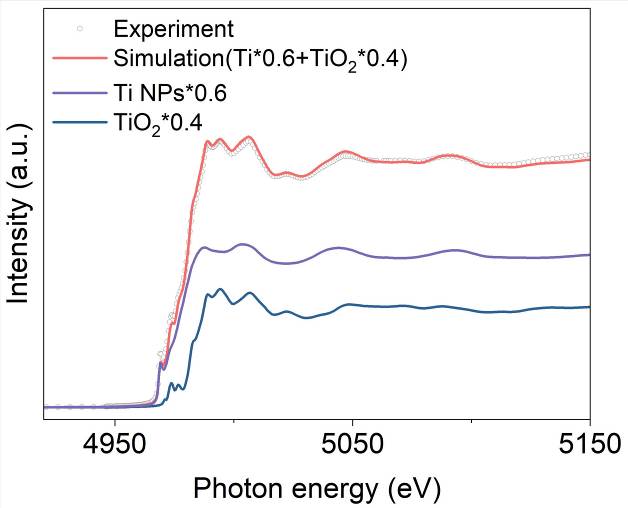


**Fig. S7** Linear combination fitting of Ti K-edge XANES spectra for Cu-TiO_2_/Ti sample.


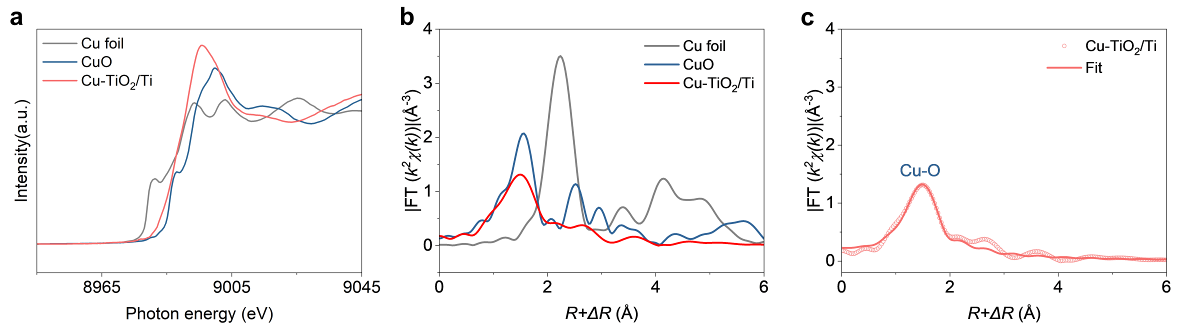


**Fig. S8 a** Cu K-edge XANES spectra for Cu foil, CuO, and Cu-TiO_2_/Ti. **b** Cu K-edge *k*^2^-weighted Fourier transform spectra for Cu foil, CuO, and Cu-TiO_2_/Ti. **c** Cu K-edge *k*^2^-weighted Fourier transform spectrum and the corresponding fitting curve of Cu-TiO_2_/Ti.


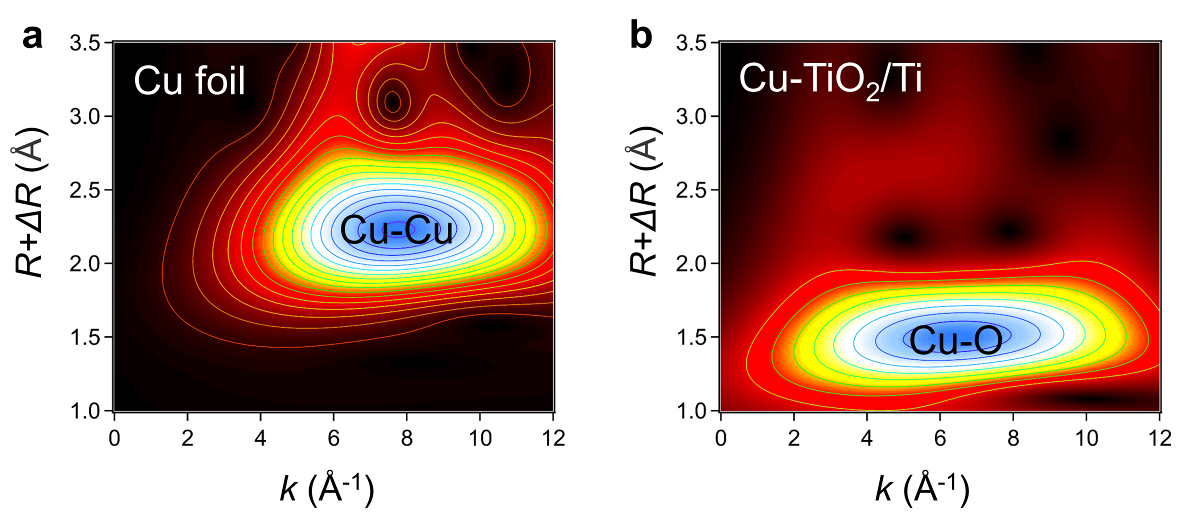


**Fig. S9** Wavelet transform of Cu K-edge EXAFS data for **a** Cu foil and **b** Cu-TiO_2_/Ti.


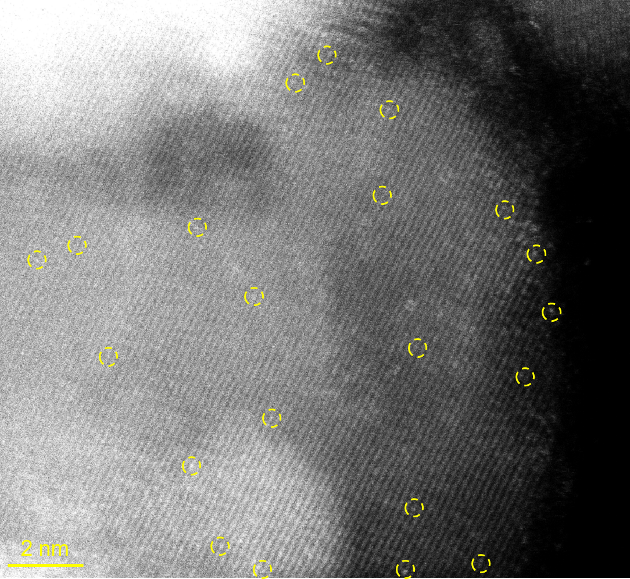


**Fig. S10** STEM-HAADF image of Cu-TiO_2_/Ti.


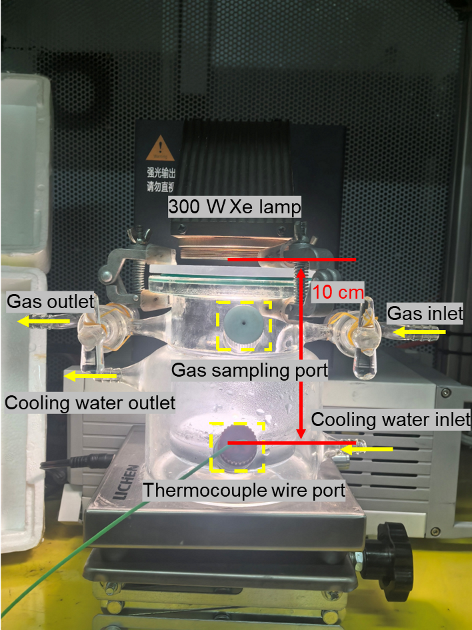


**Fig. S11** Photograph of the reaction apparatus.


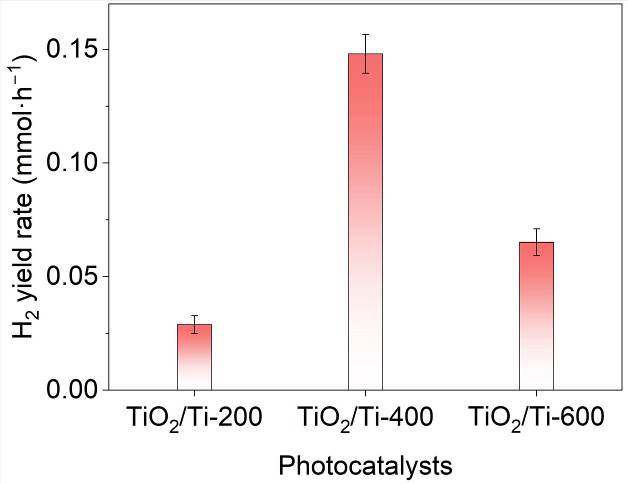


**Fig. S12** H_2_ yield rates over TiO_2_/Ti-200, TiO_2_/Ti, and TiO_2_/Ti-600 samples, respectively (Light intensity: 500 mW/cm^2^).

Note: TiO_2_/Ti-200 and TiO_2_/Ti-600 were synthesized by annealing Ti NPs at 200 ºC and 600 ºC for 1 h under the atmosphere of air, respectively.


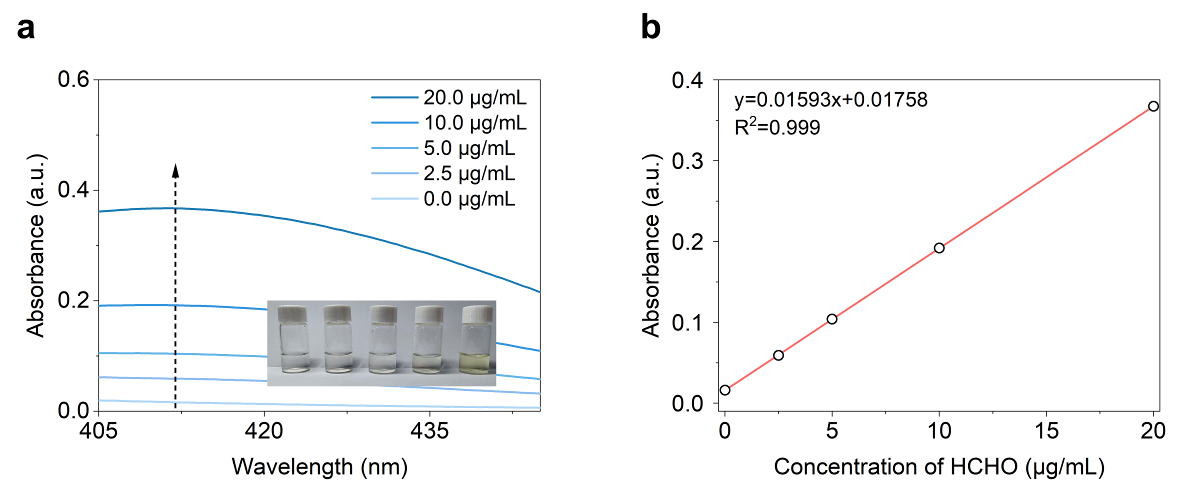


**Fig. S13** **a** The UV-vis absorption spectra of HCHO solution with different concentrations. **b** The standard curve for HCHO detection.


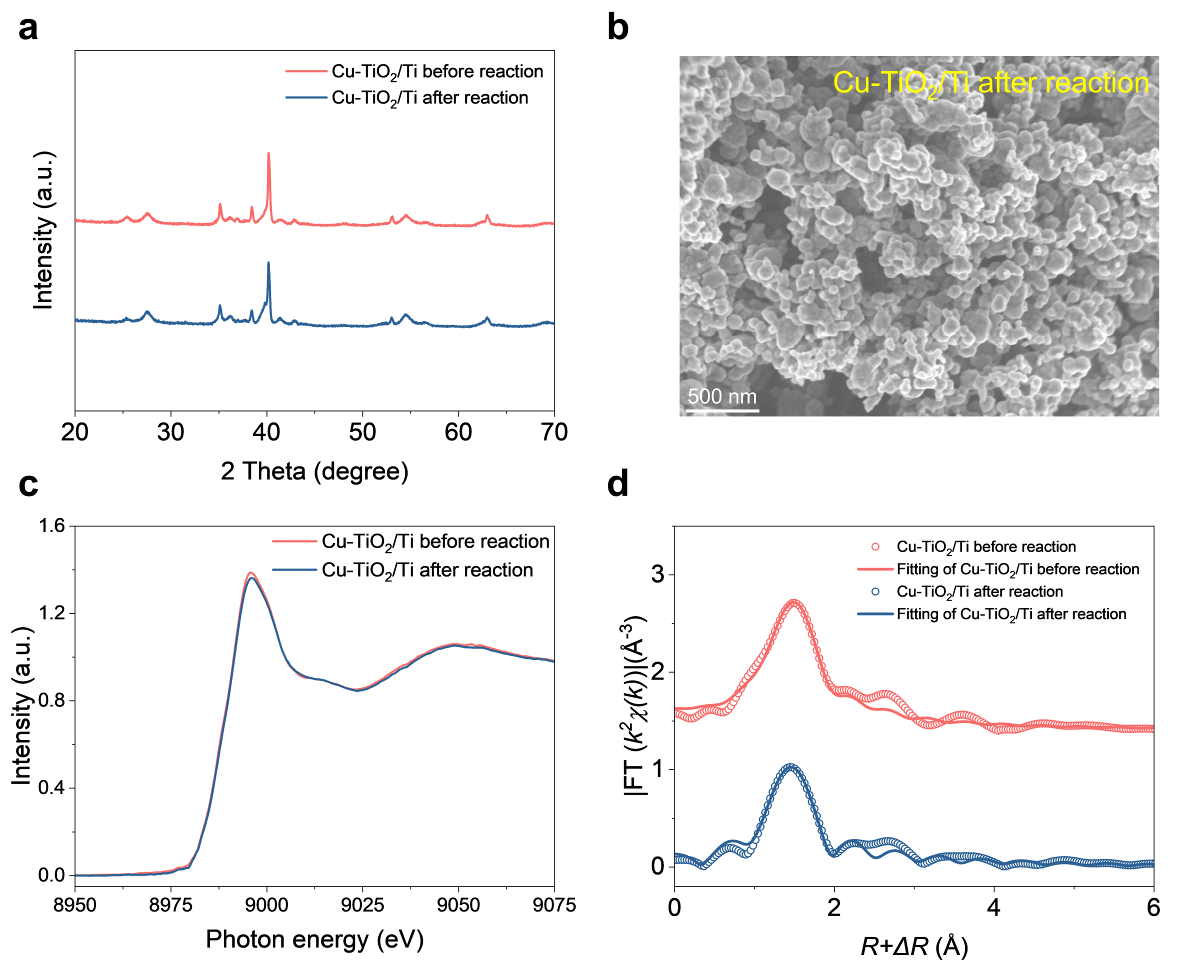


**Fig. S14 a** XRD patterns of Cu-TiO_2_/Ti before reaction and Cu-TiO_2_/Ti after reaction. **b** SEM image of Cu-TiO_2_/Ti after reaction. **c** Cu K-edge XANES spectra for Cu-TiO_2_/Ti before reaction and Cu-TiO_2_/Ti after reaction samples. **d** Cu K-edge *k*^2^-weighted Fourier transform spectra for Cu-TiO_2_/Ti before reaction and Cu-TiO_2_/Ti after reaction.


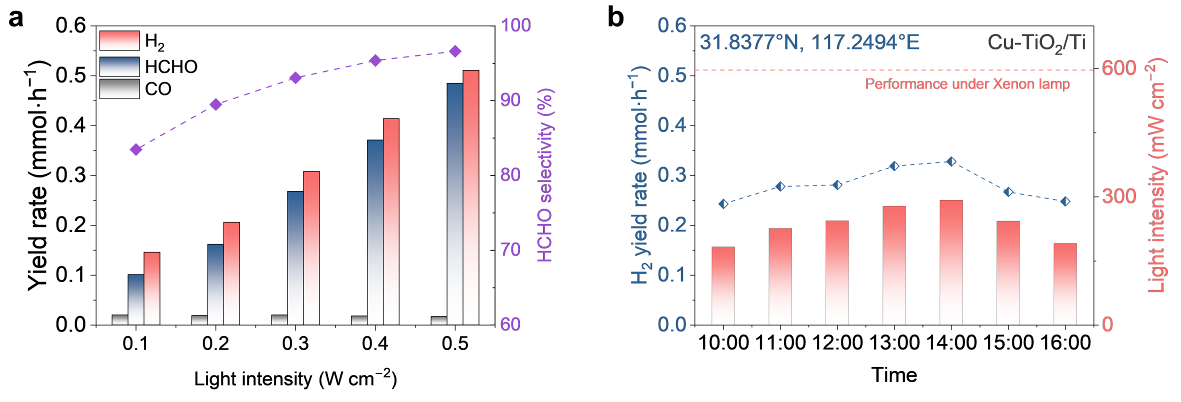


**Fig. S15** **a** The yield rates of H_2_, HCHO, and CO over Cu-TiO_2_/Ti under different light intensity. **b** H_2_ yield rates over Cu-TiO_2_/Ti under actual sunlight illumination.


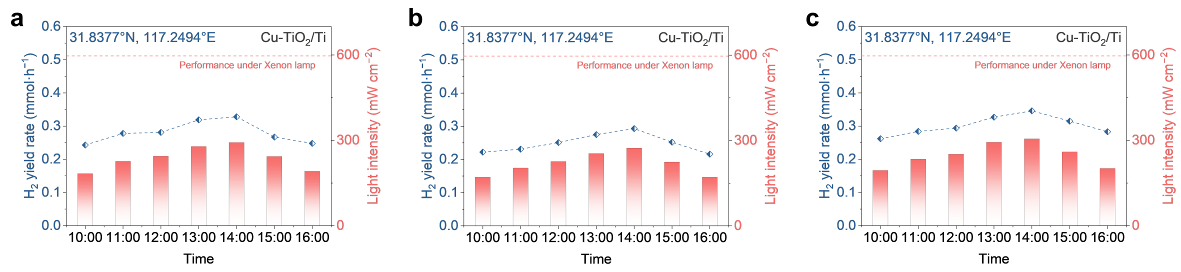


**Fig. S16** H_2_ yield rates over Cu-TiO_2_/Ti under actual sunlight illumination from three independent experiments for three days.


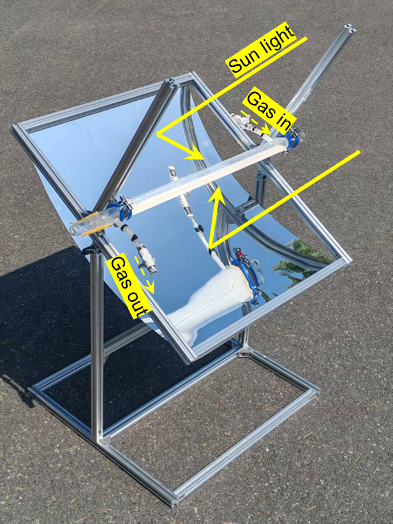


**Fig. S17** Photocatalytic reaction apparatus under natural sunlight.


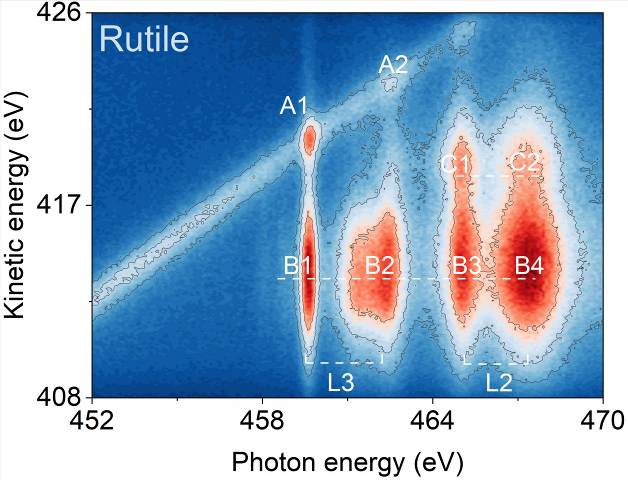


**Fig. S18** The Ti RAS spectrum of Rutile.

Note:

A1: The A1 process corresponds to a core-level electron of 2*p*_3/2_ orbital being excited into the unoccupied t_2g_ state of the 3d orbital by incident X-rays. The excited electron subsequently relaxes back to fill the 2*p*_3/2_ core hole, and the relaxation energy is transferred to the occupied 3*p* orbital, resulting in an electron of 3*d* orbital being excited above the vacuum level. This process is classified as a participator-type Auger decay.

A2: The A2 process corresponds to a core-level electron of 2*p*_3/2_ orbital being excited into the unoccupied e_g_ state of the 3*d* orbital by incident X-rays. The excited electron subsequently relaxes back to fill the 2*p*_3/2_ core hole, and the relaxation energy is transferred to the occupied 3*p* orbital, resulting in an electron of 3*d* orbital being excited above the vacuum level. This process is classified as a participator-type Auger decay.

B1: The B1 process corresponds to a core-level electron of 2*p*_3/2_ orbital being excited into the unoccupied t_2g_ state of the 3*d* orbital by incident X-rays. Subsequently, an electron from the occupied 3*p* orbital relaxes back to fill the 2*p*_3/2_ core hole, and the relaxation energy is transferred to an occupied valence-band orbital, ejecting an Auger electron above the vacuum level. This is a spectator-type Auger decay.

B2: The B2 process corresponds to a core-level electron of 2*p*_3/2_ orbital being excited into the unoccupied e_g_ state of the 3*d* orbital by incident X-rays. Subsequently, an electron from the occupied 3*p* orbital relaxes back to fill the 2*p*_3/2_ core hole, and the relaxation energy is transferred to an occupied valence-band orbital, ejecting an Auger electron above the vacuum level. This is a spectator-type Auger decay.

B3: The B3 process corresponds to a core-level electron of 2*p*_1/2_ orbital being excited into the unoccupied t_2g_ state of the 3*d* orbital by incident X-rays. The hole of 2*p*_1/2_ orbital undergoes a Coster-Kronig transition to 2*p*_3/2_ orbital. Subsequently, an electron from the occupied 3*p* orbital relaxes back to fill the 2*p*_3/2_ core hole, and the relaxation energy is transferred to an occupied valence-band orbital, ejecting an Auger electron above the vacuum level. This is a spectator-type Auger decay.

B4: The B4 process corresponds to a core-level electron of 2*p*_1/2_ orbital being excited into the unoccupied e_g_ state of the 3*d* orbital by incident X-rays. The hole of 2*p*_1/2_ orbital undergoes a Coster–Kronig transition to 2*p*_3/2_ orbital. Subsequently, an electron from the occupied 3*p* orbital relaxes back to fill the 2*p*_3/2_ core hole, and the relaxation energy is transferred to an occupied valence-band orbital, ejecting an Auger electron above the vacuum level. This is a spectator-type Auger decay.

C1: The C1 process corresponds to a core-level electron of 2*p*_1/2_ orbital being excited into the unoccupied t_2g_ state of the 3*d* orbital by incident X-rays. Subsequently, an electron from the occupied 3*p* orbital relaxes back to fill the 2*p*_1/2_ core hole, and the relaxation energy is transferred to an occupied valence-band orbital, ejecting an Auger electron above the vacuum level. This is a spectator-type Auger decay.

C2: The C2 process corresponds to a core-level electron of 2*p*_1/2_ orbital being excited into the unoccupied e_g_ state of the 3*d* orbital by incident X-rays. Subsequently, an electron from the occupied 3*p* orbital relaxes back to fill the 2*p*_1/2_ core hole, and the relaxation energy is transferred to an occupied valence-band orbital, ejecting an Auger electron above the vacuum level. This is a spectator-type Auger decay.


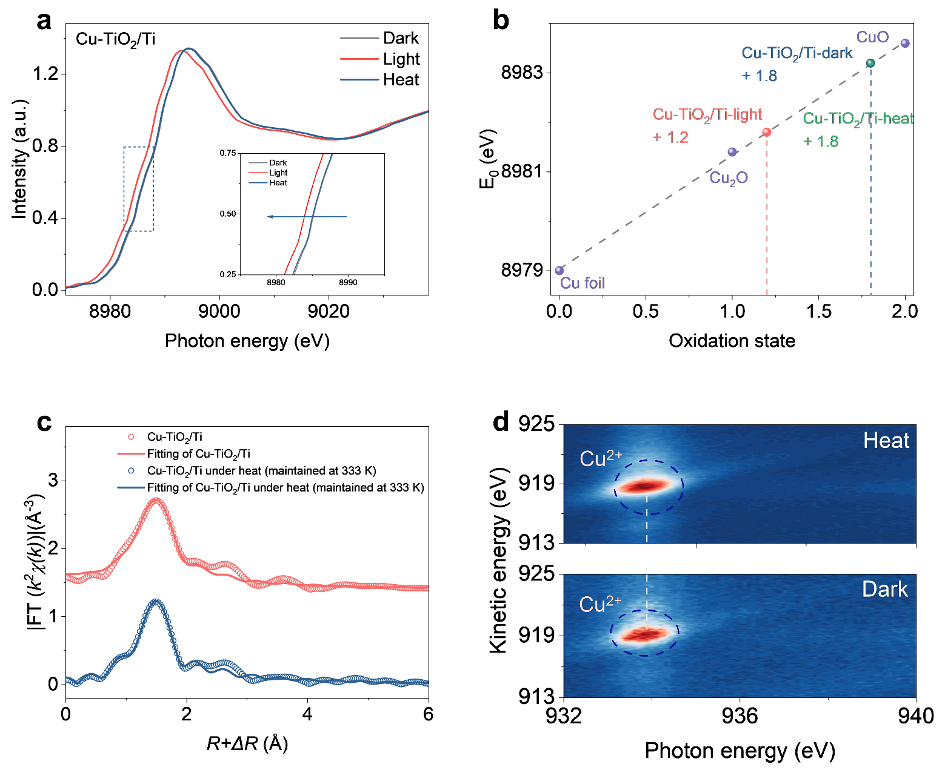


**Fig. S19 a** *In situ* XANES spectra for Cu-TiO_2_/Ti sample. **b** Average Cu oxidation state determination in dark, light and heat (maintained at 333 K) of Cu-TiO_2_/Ti, using the Cu K-edge energy shift of Cu foil, Cu_2_O, and CuO. **c** Cu K-edge *k*^2^-weighted Fourier transform spectra for Cu-TiO_2_/Ti and Cu-TiO_2_/Ti under heat (maintained at 333 K). **d** *In situ* Cu RAS spectra of Cu-TiO_2_/Ti in dark and heat (maintained at 333 K).


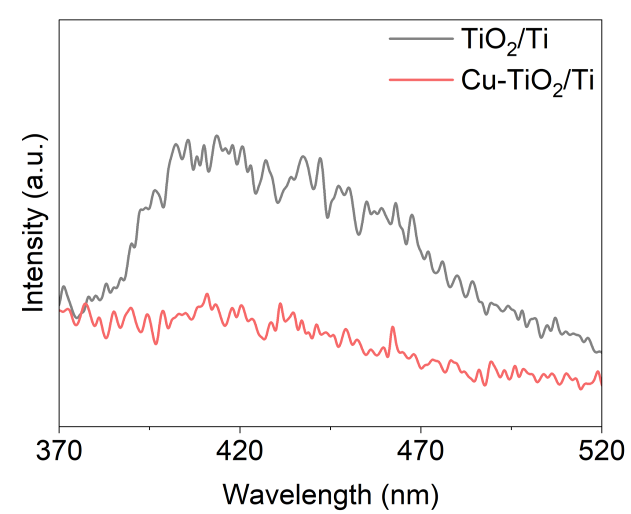


**Fig. S20** PL spectra of TiO_2_/Ti and Cu-TiO_2_/Ti samples.


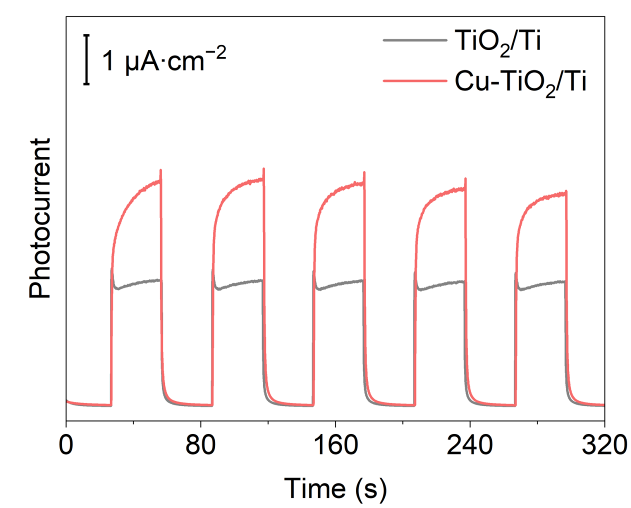


**Fig. S21** Transient photocurrent response spectra of TiO_2_/Ti and Cu-TiO_2_/Ti samples.


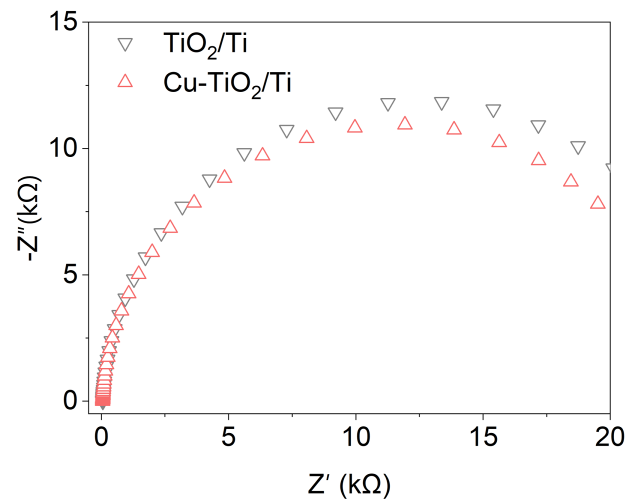


**Fig. S22**. EIS spectra of TiO_2_/Ti and Cu-TiO_2_/Ti samples.


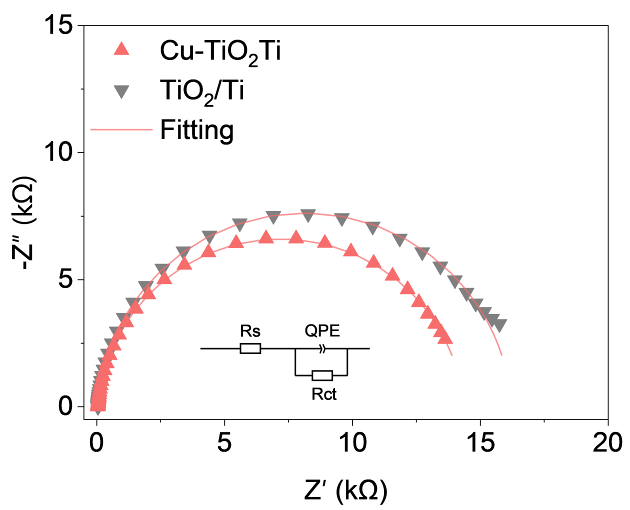


**Fig. S23** EIS spectra of TiO_2_/Ti, Cu-TiO_2_/Ti samples under light and fitting.


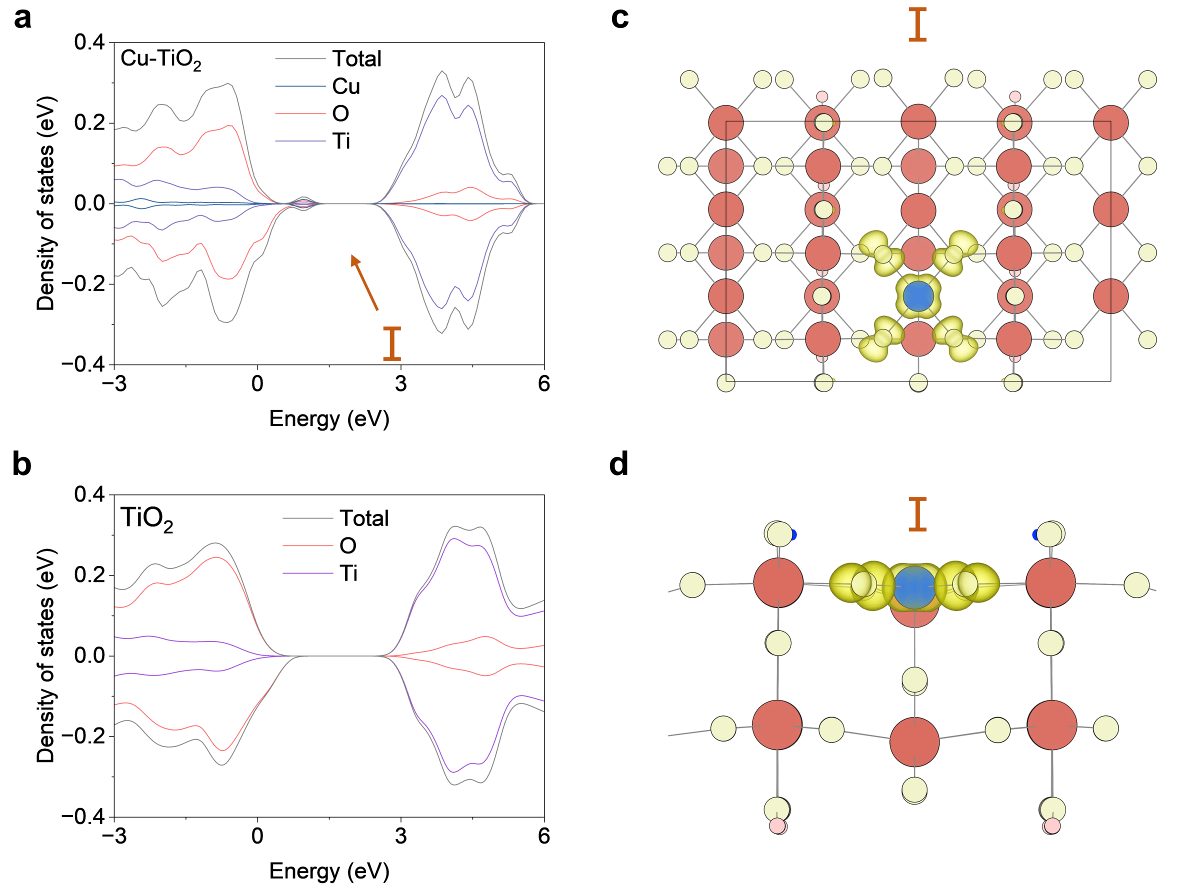


**Fig. S24** **a** Calculated electronic density of states (DOS) of Cu-TiO_2_(110). **b** Calculated electronic density of states (DOS) of TiO_2_(110). **c** The corresponding charge distributions of locations Ⅰ from the top view. **d** The corresponding charge distributions of locations Ⅰ from the side view. The balls in light yellow, red, and blue represent O, Ti, and Cu atoms, respectively. The yellow and cyan colors represent electron accumulation and depletion, respectively.


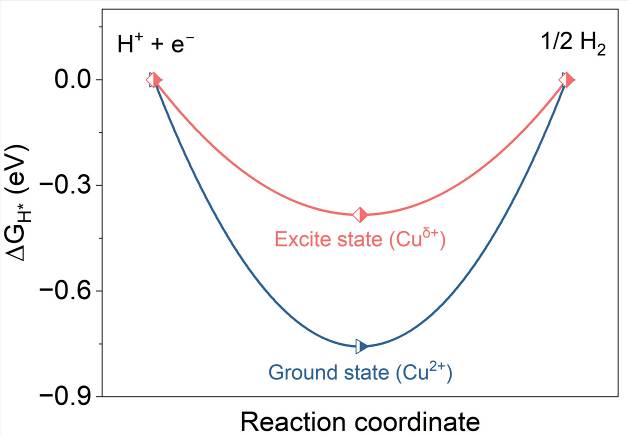


**Fig. S25** H_2_ evolution reaction energy barriers over Cu site in ground and excited states.


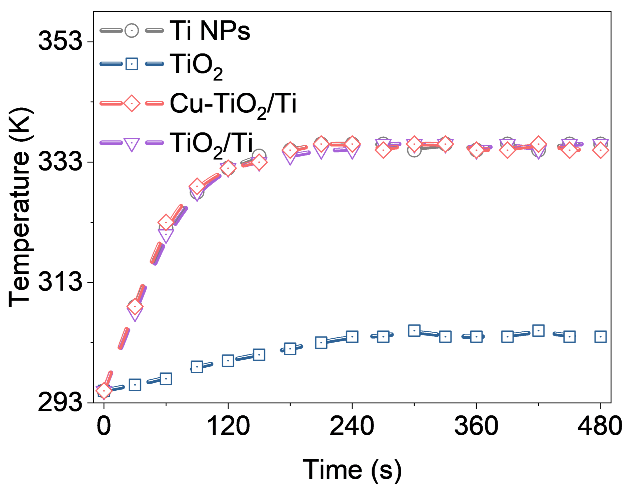


**Fig. S26** The temperature of the liquid during the lighting process over Ti NPs, Cu-TiO_2_/Ti, TiO_2_/Ti and TiO_2_, respectively.


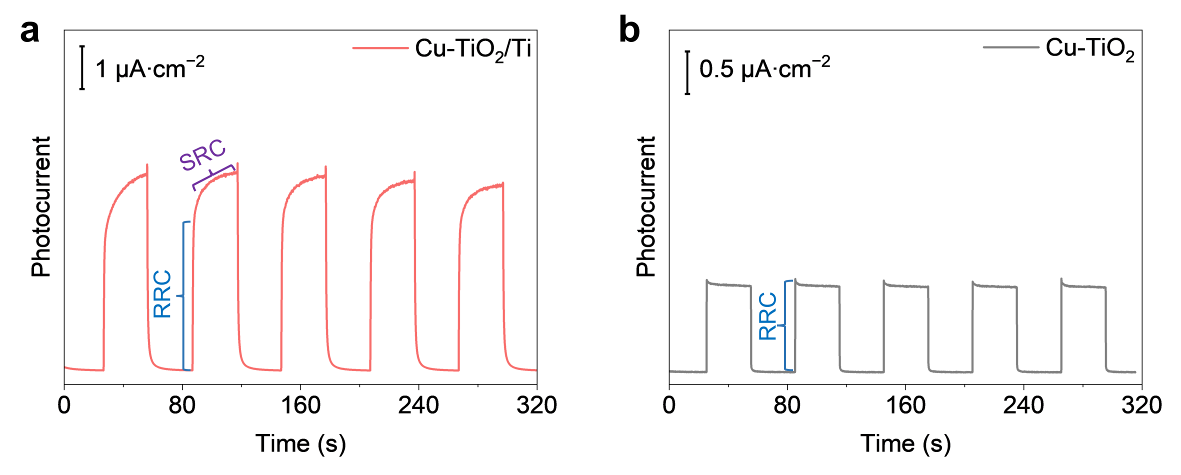


**Fig. S27** **a** Transient photocurrent response spectra of Cu-TiO_2_/Ti sample. **b** Transient photocurrent response spectra of Cu-TiO_2_ sample.


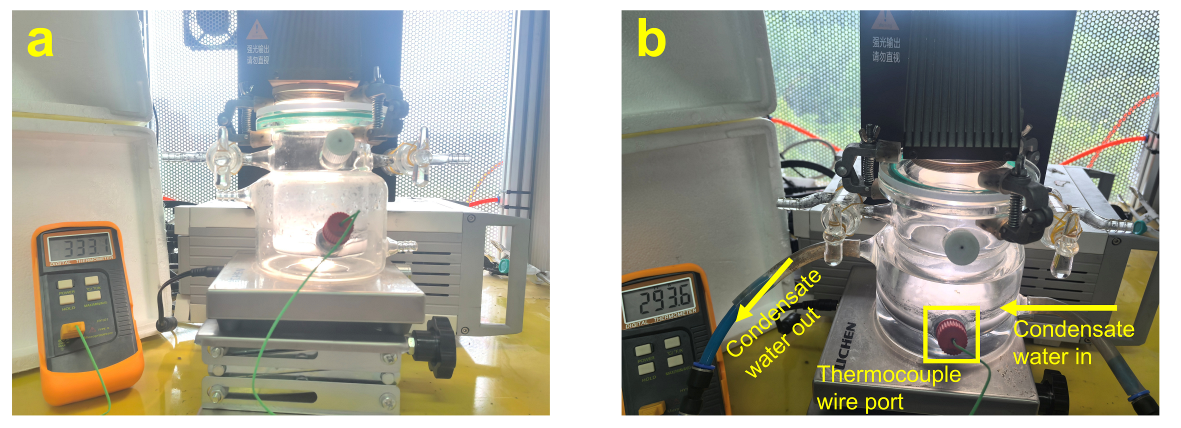


**Fig.S28** Photograph of Temperature Measurement Using a Contact Thermocouple under Practical Reaction Conditions.

**Note:** the temperature of the reaction solution under illumination was monitored using a calibrated K-type thermocouple probe, which was directly immersed in the liquid phase to ensure accurate and real-time temperature recording. The thermocouple was connected to a digital temperature acquisition system with an estimated measurement accuracy of ±0.5 °C.


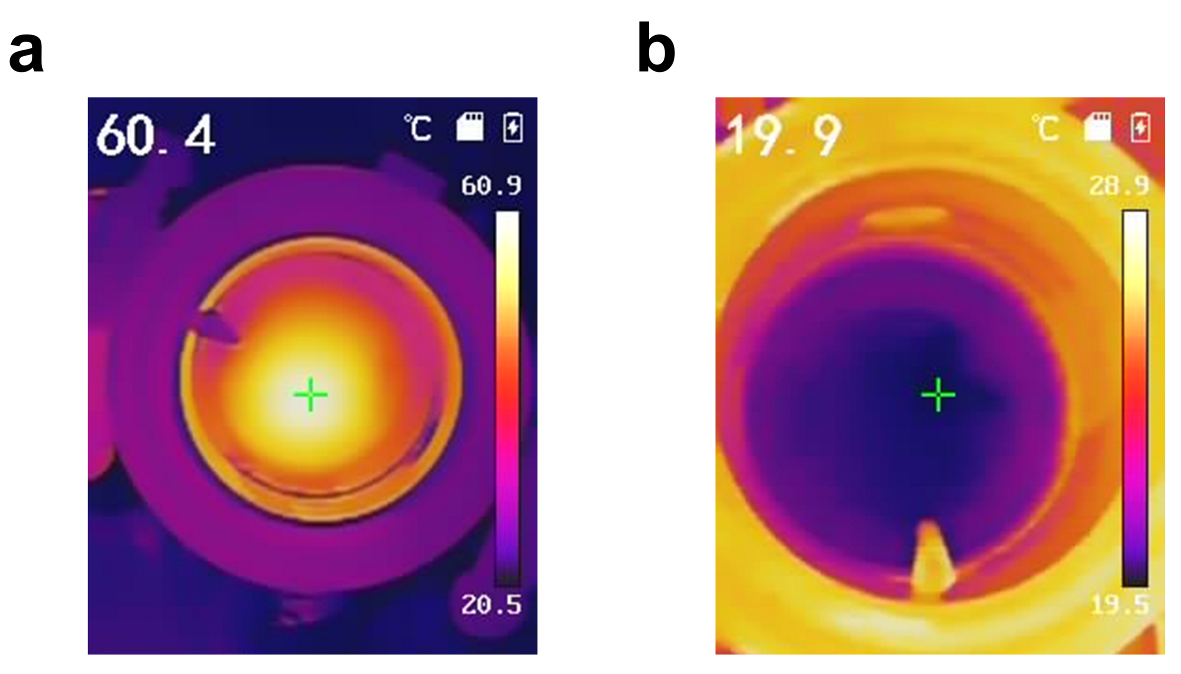


**Fig. S29** Photograph of Temperature Measurement Using an Infrared Thermometer under Practical Reaction Conditions.


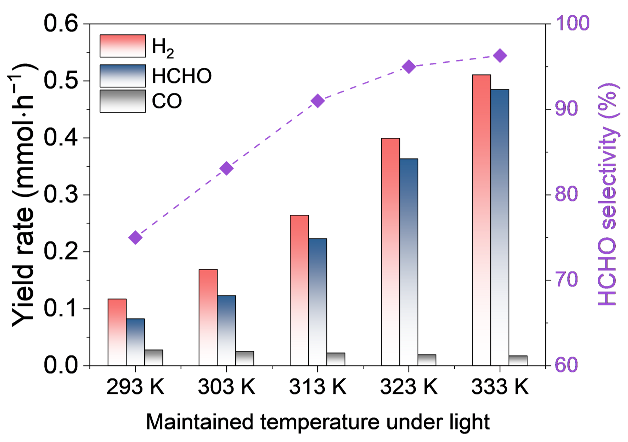


**Fig. S30** The yield rates of H_2_, HCHO, and CO over Cu-TiO_2_/Ti under different reaction temperatures with light (Light intensity: 500 mW/cm^2^).


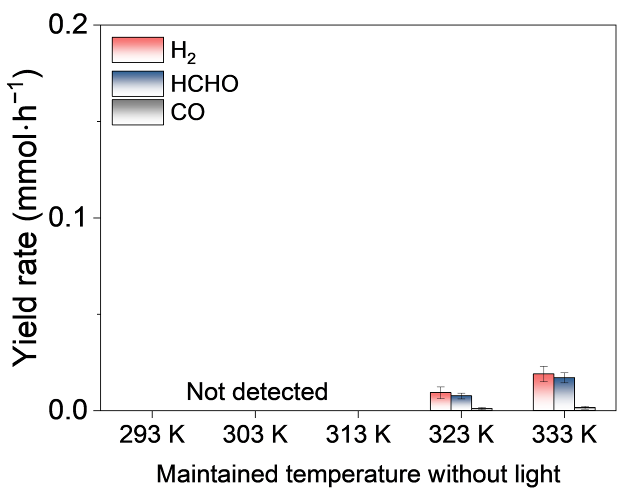


**Fig. S31** The yield rates of H_2_, HCHO, and CO over Cu-TiO_2_/Ti under different reaction temperatures without light over Cu-TiO_2_/Ti.


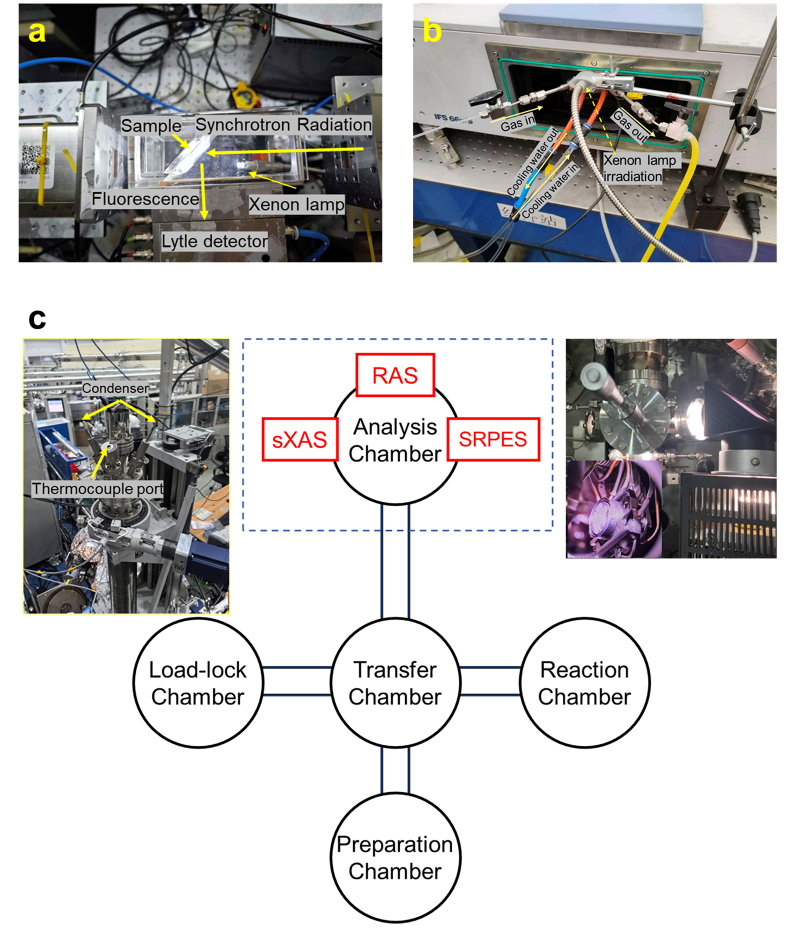


**Fig. S32 a** Photograph of the *in situ* XAFS setup. **b** Photograph of the *in situ* DRIFTS setup. **c** Photograph of the *in situ* RAS and SRPES detection principle.


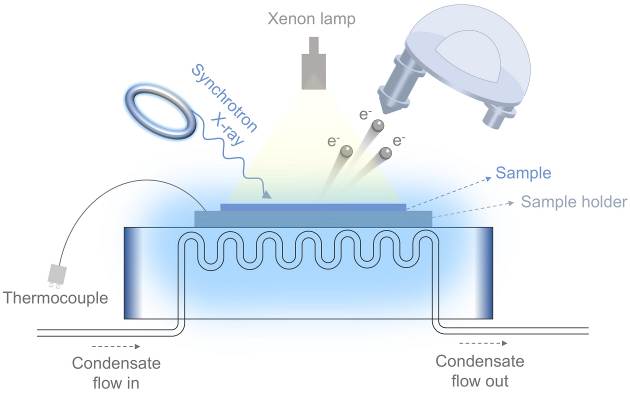


**Fig. S33** The schematic diagram of the *in situ* SRPES detection principle.


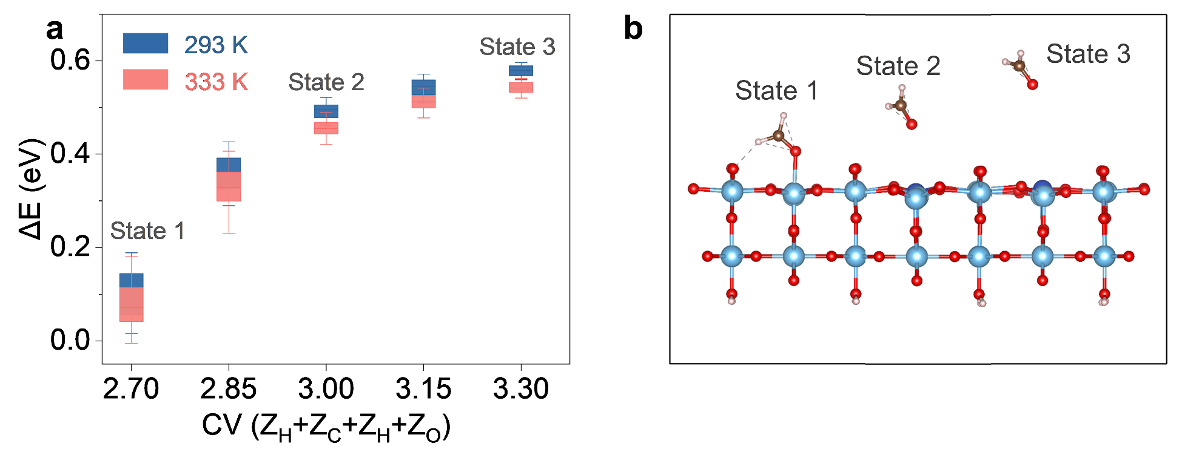


**Fig. S34** **a** Potential-dependent free energies of the HCHO desorption process over Cu-TiO_2_(110) model surface at 333 K and 293 K, respectively. Δ*E*(ζ_a_, ζ_b_) is the free energy difference between two reaction coordinates. (ζ_a_ and ζ_b_) and F(ζ) is the averaged constrained force. **b** Theoretical models of HCHO desorption over Cu-TiO_2_(110) model under different collective variable conditions.


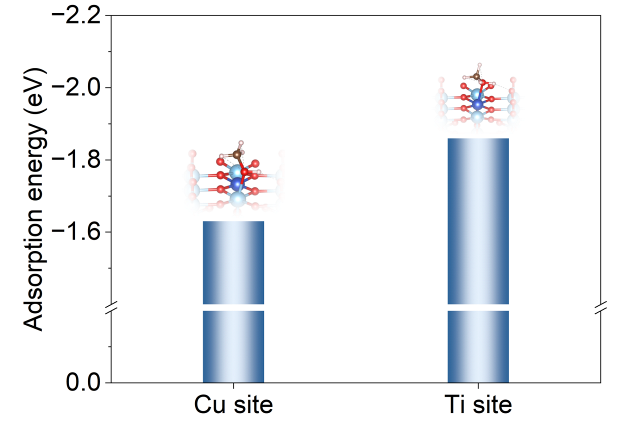


**Fig. S35** Adsorption energy calculations of CH_3_OH molecule at Cu and Ti sites, respectively.

**Table S1** The Cu content of Cu-TiO_2_/Ti before and after the cycling test.

| Catalyst | Cu content before the cycling test | | Cu content after the cycling test |
| --- | --- | --- | --- |
| Cu-TiO_2_/Ti | | 0.45 wt.% | 0.43 wt% |

**Table S2 EXAFS fitting parameters at the Cu K-edge for various samples (*Ѕ_0_^2^*=0.850).**

| Sample | Shell | *N^a^* | *R*(Å)*^b^* | *σ*^2^(Å^2^)*^c^* | Δ*E_0_* (eV)*^d^* | *R* factor |
| --- | --- | --- | --- | --- | --- | --- |
| Cu foil | Cu-Cu | 12.0 | 2.55 | 0.0056 | 4.5 | 0.0019 |
| Cu-TiO_2_/Ti | Cu-O | 4.7 | 1.94 | 0.0094 | 5.9 | 0.0007 |
| Cu-TiO_2_/Ti after reaction | Cu-O | 4.5 | 1.93 | 0.0082 | -4.5 | 0.0009 |
| Cu-TiO_2_/Ti  under heat | Cu-O | 4.7 | 1.95 | 0.0104 | 0.3 | 0.0006 |

Note: *^a^N*: coordination numbers. *^b^R*: bond distance. *^c^σ*^2^: Debye-Waller factors. *^d^ΔE*_0_: the inner potential correction. *R* factor: goodness of fit. *Ѕ_0_^2^* was set to 0.85, according to the experimental EXAFS fit of Cu foil reference by fixing coordination numbers as the known crystallographic value. The fixed parameters are underlined.

**Table S3 The yield rates of products and the selectivity of HCHO over Cu-TiO_2_/Ti**

| Catalyst | Yield rate (mmol h^−1^) | | | Selectivity of HCHO (%) |
| --- | --- | --- | --- | --- |
| Cu-TiO_2_/Ti | H_2_ | HCHO | CO | 96.6 |
|  | 0.511 | 0.485 | 0.017 |  |

Note: the selectivity of HCHO was calculated through the following equation:


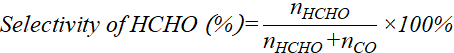


where n_HCHO_ and n_CO_ represent the amounts of substance generated HCHO and CO, respectively.

**Table S4** The solar-convert-chemical conversion efficiency (SCC) over Cu-TiO_2_/Ti

| Catalyst | SCC |
| --- | --- |
| Cu-TiO_2_/Ti | 0.21% |

**Note:** The solar-convert-chemical conversion efficiency (SCC) was calculated based on the overall Gibbs free energy change of the methanol dehydrogenation reaction:


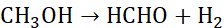


The efficiency was defined as:


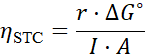


where
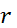
is the H_2_ production rate (mol·s⁻^1^),
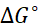
 is the standard Gibbs free energy change of the reaction (63.5 kJ·mol⁻^1^),
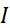
 is the incident light intensity (100 mW·cm⁻^2^), and
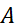
 is the irradiation area.

**Table S5 Apparent quantum yield (AQY) of the reaction over Cu-TiO_2_/Ti and TiO_2_/Ti**

| Catalyst | AQY(365 nm) |
| --- | --- |
| Cu-TiO_2_/Ti | 15.9 % |
| TiO_2_/Ti | 4.3 % |

**Note:** the AQY was calculated through the following equation:


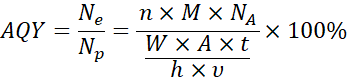


where *N_e_* and *N_p_* represent the number of reacted electrons and incident photons, respectively, *n* is the number of electrons transferred during the generation of H_2_, *M* is the molar number of H_2_, and *N_A_* is Avogadro's constant. *W, A, t, h,* and *ν* are the incident light intensity, irradiation area, irradiation time, Planck constant, and incident light frequency, respectively.

**Table S6** The summary of the reported performance of H_2_ production from photocatalytic CH_3_OH activation on representative photocatalysts

| Catalysts | Reaction conditions | H_2_ yield  (mmol h^−1^) | Stability | Ref. |
| --- | --- | --- | --- | --- |
| 0.5%Cu-TiO_2_/Ti | 333 K  300 W Xe lamp | 0.511 | 102 h | This  work |
| 0.75%Cu/TiO_2_ | Xe lamp (MAX-302) | 0.083 | 20 h (4 cycles) | [S1] |
| Cu-THS | Xe lamp | 0.063 | 72 h (6 cycles) | [S2] |
| 0.5%2D-2D MoS_2_/TiO_2_ | 300 W Xe lamp | 0.215 | 9 h (3 cycles) | [S3] |
| TiO_2_-UV100 | UV(A)-Vis illumination  (60 mW cm^-2^) | 0.3 | 3 h | [S4] |
| 0.5%Ni/TiO_2_ | UV SB-100P/F (365 nm) | 0.202 | 3 h | [S5] |
| 3%Li/TiO_2_ | Xe lamp | 0.052 | 48 h (12 cycles) | [S6] |
| Ni-ZIS NCs | 365 nm LED | 0.141 | 22 h | [S7] |
| 2%Pt/TiO_2_ | 300 W Xe lamp | 0.450 | 24 h | [S8] |
| Pt/C_3_N_4_ | 300 W Xe lamp | 0.22 | 10 h | [S9] |
| Pt/(CNT-TiO_2_)ox-473 | Heraeus TQ 150 medium pressure mercury vapor lamp | 0.25 | 2 h | [S10] |
| C-0.8Pt/TiO_2_ | 300 W Xe lamp | 0.267 | 18 h | [S11] |
| FePS_3_-TiO_2_ | 350 W Xe lamp | 0.0061 | 9 h | [S12] |
| Cu_2_O/TiO_2_ | 300 W Xe lamp | 0.1523 | 12 h | [S13] |
| MoS_2_/TiO_2_ | 365 LED | 0.122 | 12 h | [S14] |

**Table S7** Quantitative Peak Area Ratio of A1 (Participant Decay) and B1 (Spectator Decay) in the Ti RAS Spectrum.

| Catalysts | A1 | B1 |
| --- | --- | --- |
| TiO_2_/Ti | 14.4% | 85.6% |
| Cu-TiO_2_/Ti | 7.5% | 92.5% |

**Table S8** The light intensity and sample-to-Xenon lamp distance in photocatalytic experiments and different *in situ* characterization measurements.

| Application | Sample-to-Xenon lamp distance | Light intensity at the sample |
| --- | --- | --- |
| Photocatalytic experiments | 10 cm | 500 mW/cm^2^ |
| *In situ* DRIFTS | 2 cm | 500 mW/cm^2^ |
| *In situ* XAFS | 3 cm | 500 mW/cm^2^ |
| *In situ* RAS/SRPES | ～20 cm | ～500 mW/cm^2^ |

Note: During the *in situ* experiments, the light intensity at the reaction system was maintained at 500 mW/cm^2^ by adjusting the lamp current, as measured with an optical power meter (MC-PM100B, Beijing Merry Change Technology Co., Ltd.). As for the *in situ* RAS and *in situ* SRPES measurements, the sample was located inside a vacuum chamber, making it impossible to directly measure the actual light intensity at the sample position. We therefore recorded the distance from the Xe lamp to the sample (20 cm) and adopted the lamp current that, under ambient conditions, yields 500 mW/cm^2^ at a distance of 20 cm. The influence of the window medium was also taken into account to ensure that the light intensity at the sample position was as close as possible to 500 mW/cm^2^.

**Table S9** Quantitative Peak Area Ratio of Cu^+^ and Cu^2+^ in the Cu RAS Spectrum.

| Status | Cu^2+^ | Cu^+^ |
| --- | --- | --- |
| Light | 59.0% | 41.0% |
| Dark | ~100% | - |
| Heat | ~100% | - |

**Table S10** The EIS fitting data of TiO_2_/Ti, Cu-TiO_2_/Ti samples under light

| Sample | Rs (solution: Ω) | Rct (catalyst: Ω) | QPE-Q | QPE-n |
| --- | --- | --- | --- | --- |
| Cu-TiO_2_/Ti | 44.98 | 14308 | 8.9298E-6 | 0.94933 |
| TiO_2_/Ti | 48.97 | 16180 | 8.0095E-6 | 0.96183 |

Note: The Rs represents the series resistance at the interface between the photoelectrode material and FTO substrate, while Rct corresponds to charge-transfer resistance within catalysts. QPE element was introduced to describe the non-ideal capacitive behavior arising from surface heterogeneity and roughness.

**Table S11** An analysis of the photothermal and photochemical contribution

| Status | H_2_ | | HCHO |
| --- | --- | --- | --- |
| Thermal catalysis @293 K (mmol h^−1^) | -- | -- | |
| Thermal catalysis @333 K (mmol h^−1^) | 0.019 | 0.017 | |
| Light catalysis @ 293 K (mmol h^−1^) | 0.117 | 0.082 | |
| Light catalysis @333 K (mmol h^−1^) | 0.511 | 0.485 | |
| Photothermal contribution (mmol h^−1^) | 0.019 | 0.017 | |
| Photochemical contribution (mmol h^−1^) | 0.117 | 0.082 | |
| Synergistic contribution of photothermal and photochemical effects (mmol h^−1^) | 0.375 | 0.386 | |
| Photothermal contribution | 3.7% | 3.5% | |
| Photochemical contribution | 22.9% | 16.9% | |
| Synergistic contribution of photothermal and photochemical effects | 73.4% | 79.6% | |

**Table S12** Quantitative relative peak areas of the *in situ* C 1s SRPES spectra.

| Light + condensate  (maintained at 293 K) | CO_x_* | CH_2_O* | CH_3_O* | CH_3_OH* |
| --- | --- | --- | --- | --- |
| 5 min | 4.31% | 6.98% | 26.63% | 62.08% |
| 15 min | 10.76% | 10.50% | 29.24% | 49.50% |
| 30 min | 17.55% | 13.45% | 30.92% | 38.08% |
| Light  (maintained at 333 K) | CO_x_* | CH_2_O* | CH_3_O* | CH_3_OH* |
| 5 min | 9.59% | 24.98% | 27.73% | 37.70% |
| 15 min | 10.41% | 25.20% | 32.69% | 31.70% |
| 30 min | 9.02% | 50.02% | 12.49% | 28.47% |

**Supplementary References**

1. B.-H. Lee, S. Park, M. Kim, A.K. Sinha, S.C. Lee et al., Reversible and cooperative photoactivation of single-atom Cu/TiO_2_ photocatalysts. Nat. Mater. **18**(6), 620–626 (2019). <https://doi.org/10.1038/s41563-019-0344-1>
2. L. Liu, Y. Sun, Z. Ma, Q. Liu, R. Zhang et al., Vacancy-induced symmetry breaking in titanium dioxide boosts the photocatalytic hydrogen production from methanol aqueous solution. Nano Lett.. DOI:10.1021/acs.nanolett.4c03696. (2024). <https://doi.org/10.1021/acs.nanolett.4c03696>
3. Y.-J. Yuan, Z.-J. Ye, H.-W. Lu, B. Hu, Y.-H. Li et al., Constructing anatase TiO_2_ nanosheets with exposed (001) facets/layered MoS_2_ two-dimensional nanojunctions for enhanced solar hydrogen generation. ACS Catal. **6**(2), 532–541 (2016). <https://doi.org/10.1021/acscatal.5b02036>
4. T.A. Kandiel, F. Tyrsted, K.R. Petersen, N.P. Rønnau, O.A. Ljunggren et al., Photonic efficiency and mechanism of photocatalytic hydrogen production over TiO_2_: effect of particle size and surface treatment. Catal. Today **164,** 25 (2011). <https://doi.org/10.1016/j.cattod.2010.08.012>
5. W.-T. Chen, A. Chan, D. Sun-Waterhouse, J. Llorca, H. Idriss et al., Performance comparison of Ni/TiO_2_ and Au/TiO_2_ photocatalysts for H_2_ production in different alcohol-water mixtures. J. Catal. **367**, 27–42 (2018). <https://doi.org/10.1016/j.jcat.2018.08.015>
6. G. Ou, Y. Xu, B. Wen, R. Lin, B. Ge et al., Tuning defects in oxides at room temperature by lithium reduction. Nat. Commun. **9**, 1302 (2018). <https://doi.org/10.1038/s41467-018-03765-0>
7. J. Luo, C. Zhu, J. Li, J. Jin, N.E. Soland et al., Photocatalytic methanol dehydrogenation with switchable selectivity. J. Am. Chem. Soc. **147**(4), 3428–3437 (2025). <https://doi.org/10.1021/jacs.4c14413>
8. G. Jeantelot, M. Qureshi, M. Harb, S. Ould-Chikh, D.H. Anjum et al., TiO_2_-supported Pt single atoms by surface organometallic chemistry for photocatalytic hydrogen evolution. Phys. Chem. Chem. Phys. **21**(44), 24429–24440 (2019). <https://doi.org/10.1039/c9cp04470a>
9. M. Xiao, A. Baktash, M. Lyu, G. Zhao, Y. Jin et al., Unveiling the role of water in heterogeneous photocatalysis of methanol conversion for efficient hydrogen production. Angew. Chem. Int. Ed. **63**(21), e202402004 (2024). <https://doi.org/10.1002/anie.202402004>
10. C.G. Silva, M.J. Sampaio, R.R.N. Marques, L.A. Ferreira, P.B. Tavares et al., Photocatalytic production of hydrogen from methanol and TiO_2_/carbon nanotube composite materials. Int. J. Hydrogen Energy **40,** 1015–1025 (2015). <https://doi.org/10.1016/j.apcatb.2014.10.032>
11. M. Zhou, S. Xue, Q. Feng, X. Liang, W. Wu et al., Carbon layers on Pt/TiO_2_ induced dramatic promotion of photocatalytic H_2_ production: a combined experimental and computation study. Mater. Today Energy **34**, 101294 (2023). <https://doi.org/10.1016/j.mtener.2023.101294>
12. B. Xia, B. He, J. Zhang, L. Li, Y. Zhang et al., TiO_2_/FePS3 S-scheme heterojunction for greatly raised photocatalytic hydrogen evolution. Adv. Energy Mater. **12**(46), 2201449 (2022). <https://doi.org/10.1002/aenm.202201449>
13. Y. Liu, B. Zhang, L. Luo, X. Chen, Z. Wang et al., TiO_2_/Cu_2_O core/ultrathin shell nanorods as efficient and stable photocatalysts for water reduction. Angew. Chem. Int. Ed. **54**(50), 15260–15265 (2015). <https://doi.org/10.1002/anie.201509115>
14. W. Wang, S. Zhu, Y. Cao, Y. Tao, X. Li et al., Edge-enriched ultrathin MoS_2_ embedded yolk-shell TiO_2_ with boosted charge transfer for superior photocatalytic H_2_ evolution. Adv. Funct. Mater. **29**(36), 1901958 (2019). <https://doi.org/10.1002/adfm.201901958>
